# Hypertension genetic risk score is associated with burden of coronary heart disease among patients referred for coronary angiography

**DOI:** 10.1371/journal.pone.0208645

**Published:** 2018-12-19

**Authors:** Maria Lukács Krogager, Regitze Kuhr Skals, Emil Vincent R. Appel, Theresia M. Schnurr, Line Engelbrechtsen, Christian Theil Have, Oluf Pedersen, Thomas Engstrøm, Dan M. Roden, Gunnar Gislason, Henrik Enghusen Poulsen, Lars Køber, Steen Stender, Torben Hansen, Niels Grarup, Charlotte Andersson, Christian Torp-Pedersen, Peter E. Weeke

**Affiliations:** 1 Unit of Epidemiology and Biostatistics, Aalborg University Hospital, Aalborg, Denmark; 2 Department of Health Science and Technology, Aalborg University, Aalborg, Denmark; 3 Department of Cardiology, Aalborg University Hospital, Aalborg, Denmark; 4 Novo Nordisk Foundation Center for Basic Metabolic Research, Faculty of Health and Medical Sciences, University of Copenhagen, Copenhagen, Denmark; 5 Department of Cardiology, Copenhagen University Hospital Rigshospitalet, Copenhagen, Denmark; 6 Departments of Medicine, Pharmacology, and Biomedical Informatics, Vanderbilt University Medical Center, Nashville, Tennessee, United States of America; 7 Department of Cardiology, Copenhagen University Hospital, Herlev and Gentofte, Hellerup, Denmark; 8 Faculty of Health and Medical Sciences, University of Copenhagen, Copenhagen, Denmark; 9 Danish Heart Foundation, Copenhagen, Denmark; 10 The National Institute of Public Health, University of Southern Denmark, Odense, Denmark; 11 Laboratory of Clinical Pharmacology, Copenhagen University Hospital Rigshospitalet, Copenhagen, Denmark; 12 Department of Clinical Pharmacology, Bispebjerg and Frederiksberg Hospital, Copenhagen, Denmark; 13 Department of Nutrition, Exercize and Sports, Copenhagen University, Frederiksberg, Denmark; 14 Department of Cardiology, Herlev and Gentofte Hospital, Hellerup, Denmark; 15 Department of Cardiology, Bispebjerg and Frederiksberg Hospital, Denmark; Kaohsiung Medical University Hospital, TAIWAN

## Abstract

**Background:**

Recent GWAS studies have identified more than 300 SNPs associated with variation in blood pressure. We investigated whether a genetic risk score constructed from these variants is associated with burden of coronary heart disease.

**Methods:**

From 2010–2014, 4,809 individuals admitted to coronary angiography in Capital Region of Copenhagen were genotyped. We calculated hypertension GRS comprised of GWAS identified SNPs associated with blood pressure. We performed logistic regression analyses to estimate the risk of hypertension and prevalent CHD. We also assessed the severity of CHD associated with the GRS. The analyses were performed using GRS quartiles. We used the Inter99 cohort to validate our results and to investigate for possible pleiotropy for the GRS with other CHD risk factors.

**Results:**

In COGEN, adjusted odds ratios comparing the 2^nd^, 3^rd^ and 4^th^ cumulative GRS quartiles with the reference were 1.12(95% CI 0.95–1.33), 1.35(95% CI 1.14–1.59) and 1.29(95% CI 1.09–1.53) respectively, for prevalent CHD. The adjusted multinomial logistic regression showed that 3^rd^ and 4^th^ GRS quartiles were associated with increased odds of developing two(OR 1.33, 95% CI 1.04–1.71 and OR 1.36, 95% CI 1.06–1.75, respectively) and three coronary vessel disease(OR 1.77, 95% CI 1.36–2.30 and OR 1.65, 95% CI 1.26–2.15, respectively). Similar results for incident CHD were observed in the Inter99 cohort. The hypertension GRS did not associate with type 2 diabetes, smoking, BMI or hyperlipidemia.

**Conclusion:**

Hypertension GRS quartiles were associated with an increased risk of hypertension, prevalent CHD, and burden of coronary vessel disease in a dose-response pattern. We showed no evidence for pleiotropy with other risk factors for CHD.

## Introduction

Hypertension has been estimated to account for >45% of all coronary heart disease (CHD) events worldwide [1]. The relation between genetic determinants of hypertension and the development of CHD can therefore be important for understanding disease mechanisms. More than 300 single nucleotide polymorphisms (SNPs) with relation to hypertension have been identified in genome-wide association studies (GWAS) [2], [3]. The relation between these many hypertension associated SNPs and occurrence of CHD has received limited attention. A single study has indicated a dose-response relationship of increased risk of CHD associated with the number of hypertension associated SNPs [4]. There are no studies of a relation between hypertension associated SNPs and severity of CHD. Further understanding of the genetic architecture of hypertension and CHD requires interrogation of datasets where the CHD phenotype is very certain and also quantifiable.

We have for the current study used a dataset from 4,809 individuals that have been subjected to genotyping and coronary angiography enabling us determine the presence/absence of CHD with very high certainty and further to quantify the extent of disease by number of vessels affected. In this report we calculated a genetic risk score using coefficients from a prior GWAS study and analyzed the relation between the genetic risk score and both presence and severity of CHD.

## Methods

### Study populations

#### COGEN

The Copenhagen Cardiovascular Genetic study (COGEN) is a biobank that includes ~80,000 individuals admitted to six cardiology departments in the Greater Region of Copenhagen, Denmark, from 2010–2017. The present study is based on a sample of ~5,900 individuals who have had at least one coronary angiogram performed between 2010–2014 and who have been genotyped as described below and in the online supporting information.

Information on diagnostic results from angiograms was obtained from The Eastern Danish Heart Registry. Included in the Eastern Danish Heart Registry are clinical data on all patients undergoing cardiac catheterization and coronary revascularization in Eastern Denmark since 1998 [5]. The registry includes information on demographics (e.g. age, gender), risk factors and comorbidities (e.g. smoking status, diabetes, hypertension) and coronary pathology. Patients with significant CHD were defined as patients needing percutaneous coronary intervention (PCI) or coronary artery bypass surgery (CABG). Moreover, this registry also provides data on the type of clinical presentation such as ST-elevation myocardial infarction (STEMI), non-ST elevation myocardial infarction (NSTEMI), unstable (UAP) and stable angina pectoris (SAP).

We included 4,809 patients in the present study after excluding patients with missing values on one of the following variables: age, gender, smoking, BMI, history with acute myocardial infarction (AMI), coronary artery bypass surgery (CABG), peripheral artery disease (PAD), diabetes, hypertension, hyperlipidemia, stroke.

#### Inter99

To analyze whether the hypertension GRS is associated with CHD also in a population based cohort (i.e. corresponding to a primary preventive setting), we used the Inter99 population, a cohort from 11 municipalities in the south-western part of Copenhagen. This population has been described in detail previously [6]–[8]. In brief, the Inter99 study is a randomized, non-pharmacological intervention study for prevention of ischemic heart disease on more than 13,000 individuals between 30 and 60 years randomly selected from the Civil Registration System. The study was conducted at the Research Centre for Prevention and Health in Glostrup, Denmark (https://www.regionh.dk/rcph/population-based-epidemiology/Pages/The-Inter99-Study.aspx) [6]. Participants were further prerandomized into high-intensity (90%) and low-intensity (10%) intervention groups. Baseline health examinations were attended by 6,784 (52%). At baseline all individuals received individual lifestyle counseling, with focus on smoking, use of alcohol, diet and physical activity. Participants in the high intensity groups were, additionally, offered group-based lifestyle counseling if they were evaluated at high risk for developing ischemic heart disease. Follow-up examinations after 5 years were conducted with a participation rate of 66% (n = 4,511) [7], [9]. This intervention study was of rather low intensity with examinations and counseling up to four times over five years. Individuals at high risk of ischemic heart disease, were offered six extra group-based lifestyle counseling sessions. The intervention study had no effects on ischemic heart disease, stroke and all-cause mortality after 10 years [10]. Overall, genotype information was available on 6127 individuals, but 1215 participants were excluded due to missing values on age, gender, smoking, BMI, history with AMI, diabetes, hypertension, hyperlipidemia and stroke. Hypertension was defined by the use of antihypertensive treatment or blood pressure measurements over 140/90 mm Hg at study entry. In the present study, we used the Inter99 population to investigate for pleiotropy between the hypertension GRS and other CHD risk factors defined as type 2 diabetes, BMI, hyperlipidemia, and smoking.

### Genotyping, imputation and quality control

In COGEN, genome-wide genotyping was performed using the Illumnia Infinium Human CoreExome Beadchip-24 v1.0 (Illumnia, San Diego, CA, USA) and mapped to GRCh37.p13 assembly (i.e. hg19). Genotypes were called using the Genotyping module (version 1.9.4) of GenomeStudio Software (version 2011.1, Illumina). We removed variants with missingness above 5%, and excluded individuals who had a call-rate below 95%, extreme inbreeding coefficients, mislabelled sex, were non-ethnical Danes (based on principal component analysis), or duplicates. We excluded one individual in each close family pair (Siblings, monozygotic twins or parent-offspring) pair identified by an identity by descent (IBD) analysis. In total 5,128 patients and 539,004 variants passed quality control (QC) analyses and were eligible for imputation.

Imputation was performed on the Sanger Imputation Server using haplotypes from the Haplotype Reference Consortium (HRC version r1.1) panel [11] on the subset of variants that were not significant in a Hardy Weinberg Equilibrium test (p<0.0001). Supporting information S1 File includes detailed information about genotyping, QC analyses and imputation.

Individuals from Inter99 were genotyped using the Cardio-Metabochip [12] and Human Exome BeadChip on an Illumina HiScan system (Illumina, San Diego, CA). Quality control and genotype calling have previously been described [13].

### Single nucleotide polymorphism selection and GRS

We identified 301 SNPs that reached genome-wide significance (p = 5×10^−8^) for SBP, DBP and/or PP from the largest GWAS meta-analysis to date by Hoffmann et al [3]. Of these, 12 SNPs were excluded due to a minor allele count (MAC) < 20 in our study sample (S1 Table for details). Overall, 33 SNPs were not captured by genotyping or subsequent imputation. Of these we identified relevant proxy SNPs for a total of 10 SNPs (r^2^ > 0.8 according to LDlink [14]), and 23 SNPs did not have relevant proxies (S2 Table).

We calculated two different scores where we oriented each coding allele to be the risk allele, regardless of allele frequency. In the unweighted GRS (uGRS), we assigned 1 point per risk allele assuming an additive risk model. GRS_i_ is the unweighted GRS for individual *i*, s_ij_ is the number of effect increasing alleles for the *j*’th SNP for individual *i* and N is the number of SNPs in the GRS:

$$u{GRS}_{i}=\sum_{j=1}^{N}s_{ij}$$

In the weighted GRS (wGRS), the risk alleles were weighted by the effect size (beta-estimates) of the variant reported in the relevant discovery study [3], [15], [16]. Thus, for the wGRS, we multiplied each allele with the reported effect size, after which we summed up the total value of all weighted SNPs. We then normalized by dividing by the average effect size. wGRS_i_ is the weighted GRS for individual *i*, s_ij_ is the number of effect increasing alleles for the *j*’th SNP for individual *i*, N is the number of SNPs in the GRS and β_j_ is the reported effect size for the *j*’th SNP.

$${wGRS}_{i}=\frac{N}{\sum_{j=1}^{N}\beta_{j}}\sum_{j=1}^{N}\beta_{j}s_{ij}$$

Both scores were afterwards standardized to have mean zero, and a standard deviation at one.

No correlated SNPs were found applying an LD-filter (r^2^ <0.8). SNPs and effect sizes used for weighting of the specific variants can be found in S3 Table.

### Statistical analyses

Logistic regression analysis was used to assess the relation of prevalent hypertension and CHD associated with the calculated uGRS. In order to analyze the impact of uGRS on burden of coronary vessel disease (i.e. zero-, one-, two-, or three- coronary vessel disease) we performed multinomial logistic regression with four strata of the dependent variable: no coronary vessel disease, one coronary vessel disease, two coronary vessel disease and three coronary vessel disease. The multivariable model was adjusted for age, gender, smoking, BMI, diabetes, hyperlipidemia, stroke and peripheral artery disease (PAD). We used, in the main analyses, the uGRS instead of the wGRS to ascertain the risk of prevalent hypertension and CHD as the weights derived from risk factor discovery study, where the coefficients reflect relative risks and not absolute risks that are applicable to the general population [17]. However, we also analyzed whether the wGRS followed the same trend as the uGRS and included the results in the online supporting information. We also evaluated the risk of coronary heart disease in relation to the each of the three hypertension traits (SBP, DBP and PP) individually. Finally, we ascertained whether there were associations between hypertension GRS and other risk factors including diabetes, smoking, BMI or hyperlipidemia.

The analyses were performed using u/wGRS quartiles with the lowest interval as reference. When presenting the results, we refer to these quantiles as 1^st^ (lowest), 2^nd^, 3^rd^ and 4^th^ (highest) quartiles. Age was also divided into quartiles, using the youngest as reference. No interaction between age and GRS was found regardless of the outcome (hypertension and CHD). We divided BMI into the following subgroups: underweight (<18.5 kg/m^2^), normal weight (18.5–25 kg/m^2^), overweight (25.1–30 kg/m^2^) and obese (>30 kg/m^2^) [18]. Patients with normal weight were used as reference. We used the Analysis of variance (ANOVA) to test whether there was a statistical difference in hypertension GRS in relation to number of vessel disease. Analyses were performed R statistical software (version 3.0.1, R development core team).

### Ethics

All data were de-identified prior to analyses. Written informed consent was obtained from all participants included in the present study. The ethics committee of Region North Jutland (N-20140048) approved the project and COGEN has permission from the Data Protection Agency (00916 GEH-2010-001). The Inter99 study was approved by the Scientific Ethics Committee of the Capital Region of Denmark (KA98155) and registered as clinical trial (ClinicalTrials.gov; ID-no: NCT00289237). Both studies were performed in accordance with the principles of the Declaration of Helsinki.

## Results

### Demographics (COGEN)

The characteristics of the study population undergoing coronary angiography are presented in Table 1. Median age in the population was 66.2 years [IQR: 57.6, 73.6] and 64.2% were males. Approximately 55% of the patients were known with hypertension. Moreover, 39.3% of the patients were overweight and 26.5% were obese. Median wGRS and uGRS was 398.4 [IQR: 389.5, 407.5] and 383.6 [IQR: 373.9, 393.4] respectively. Supporting information S1 and S2 Figs. illustrate boxplots of hypertension GRS distribution according to prevalent hypertension and number of affected coronary vessels, respectively. The most common causes of referral for CAG were SAP (43%), STEMI (17%), NSTEMI (9.5%), and UAP (7.3%). Overall, 53.9% of patients had significant CHD verified by CAG; one-coronary vessel disease comprised 26.6%, two-coronary vessel disease 13.9% and, three-coronary vessel disease comprised 13.4%. A total of 46.1% of patients referred for CAG did not have significant CHD.

**Table 1:** Demographics (N = 4,809).

Variable	Total
Age (median [iqr])	**66.2 [57.6, 73.6]**
Gender (male)	**3089 (64.2%)**
wGRS^a^ (median [iqr])	**398.4 [389.5, 407.5]**
wGRS^b^ (quantiles)	
1.Q.	**1259 (26.2%)**
2.Q.	**1222 (25.4%)**
3.Q.	**1187 (24.7%)**
4.Q.	**1141 (23.7%)**
uGRS^c^ (median [iqr])	**383.6 [373.9, 393.4]**
uGRS^d^ (quantiles)	
1.Q.	**1257 (26.1%)**
2.Q.	**1229 (25.6%)**
3.Q.	**1185 (24.6%)**
4.Q	**1138 (23.7%)**
No. of vessel disease	
0	**2219 (46.1%)**
1	**1277 (26.6%)**
2	**670 (13.9%)**
3	**643 (13.4%)**
Smoking	**1153 (24.0%)**
BMI^e^	
Underweight	**58 (1.2%)**
Normal range	**1588 (33.0%)**
Overweight	**1890 (39.3%)**
Obese	**1273 (26.5%)**
Hypertension	**2707 (56.3%)**
Hyperlipidemia	**2778 (57.8%)**
Diabetes	**843 (17.5%)**
Stroke	**382 (7.9%)**
Peripheral artery disease	**302 (6.3%)**
Acute myocardial infarction	**700 (14.6%)**
CABG^f^	**329 (6.8%)**
CAG^g^ referral diagnosis	
Stable angina pectoris	**2058 (43.0%)**
Unstable angina pectoris	**350 (7.3%)**
Non-STEMI	**454 (9.5%)**
STEMI	**831 (17.4%)**
Chronic heart failure	**279 (5.8%)**
Other	**817 (17.1%)**
missing	**20**

^a^wGRS- weighted genetic risk score

^b^uGRS quantiles based on the number of hypertension increasing risk alleles: 1.Q. [337, 374]; 2.Q. (374, 384]; 3.Q. (384, 394]; 4.Q.(394, 442]

^c^uGRS- unweighted genetic risk score

^d^uGRS quantiles based on the number of hypertension increasing risk alleles: 1.Q. [353, 390]; 2.Q. [390, 399]; 3.Q.[399, 408]; 4.Q. [408, 453]

^e^BMI- body mass index

^f^CABG- coronary artery bypass graft

^g^CAG- coronary angiography

ANOVA showed that hypertension GRS is overall statistically different throughout the four vessel disease strata. Generalized linear models revealed no difference in hypertension GRS in patients with one-coronary vessel disease compared to none-coronary vessel disease (S4 Table). We observed 64 SNPs that were associated with two or three of the blood pressure traits. However, we found no correlation between SBP, DBP and PP scores.

### Hypertension genetic risk score and risk of prevalent hypertension (COGEN)

We assessed the validity of the hypertension GRS in our study population. Both unadjusted and adjusted models showed a dose-response relationship between uGRS and the increased odds of hypertension. Results of these analyses can be seen in S3 Fig.

### Hypertension genetic risk score and risk of significant coronary heart disease (COGEN)

**Fig 1:**
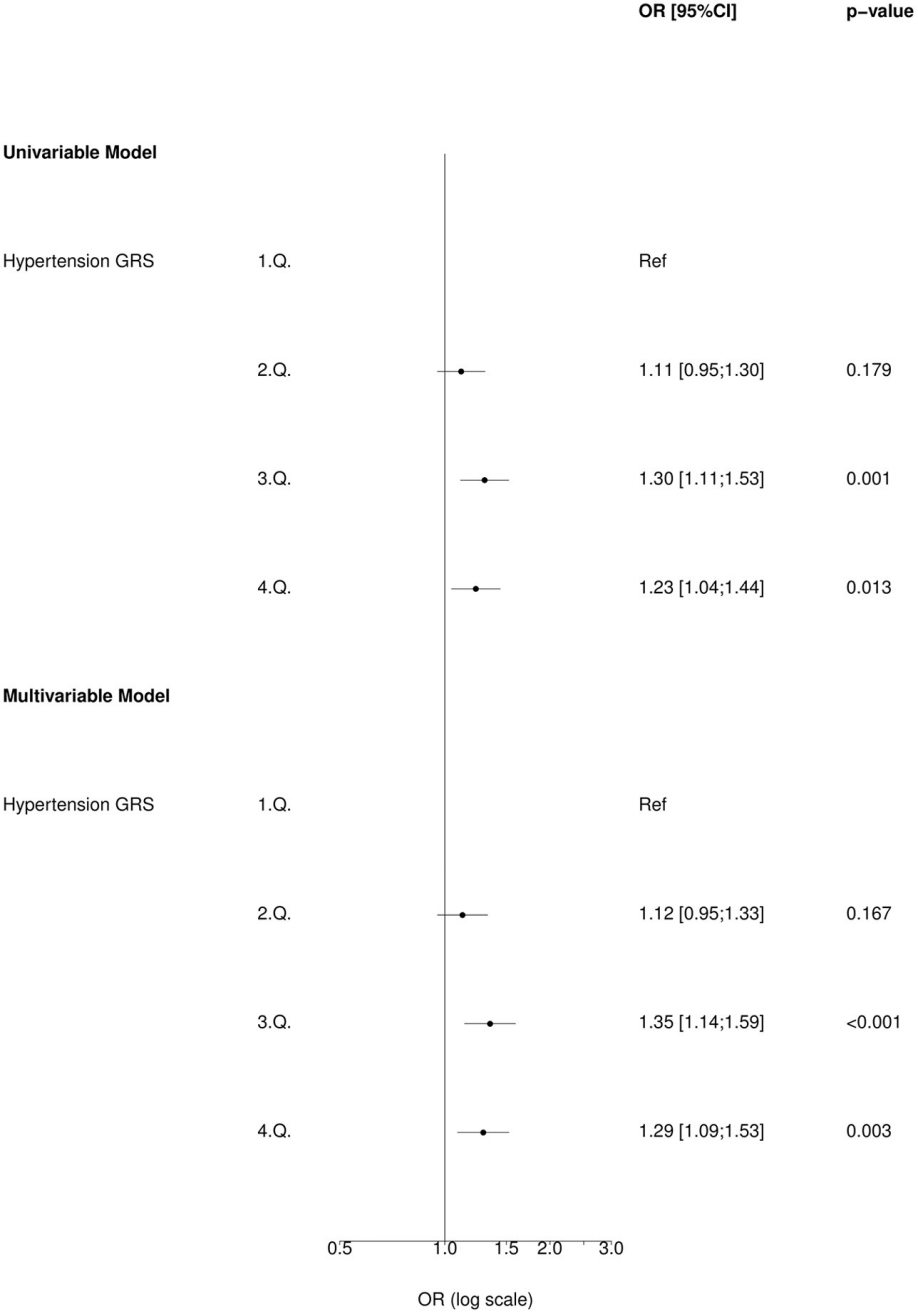
Association of blood pressure uGRS with CHD (binary outcome) in COGEN cohort (N = 4,809).

The adjusted model with significant CHD entered as the outcome variable showed that 3^rd^ and 4^th^ quartiles of the uGRS were associated with increased odds of CHD (OR 1.35, 95% CI 1.14–1.59 and OR 1.29 95% CI 1.09–1.53 respectively, Fig 1) among patients referred for CAG compared to individuals in the 1^st^ quartile. The univariable analysis showed similar results (Fig 1). We observed similar results using wGRS (S4 Fig).

### Hypertension genetic risk score and burden of significant coronary heart disease (COGEN)

**Fig 2:**
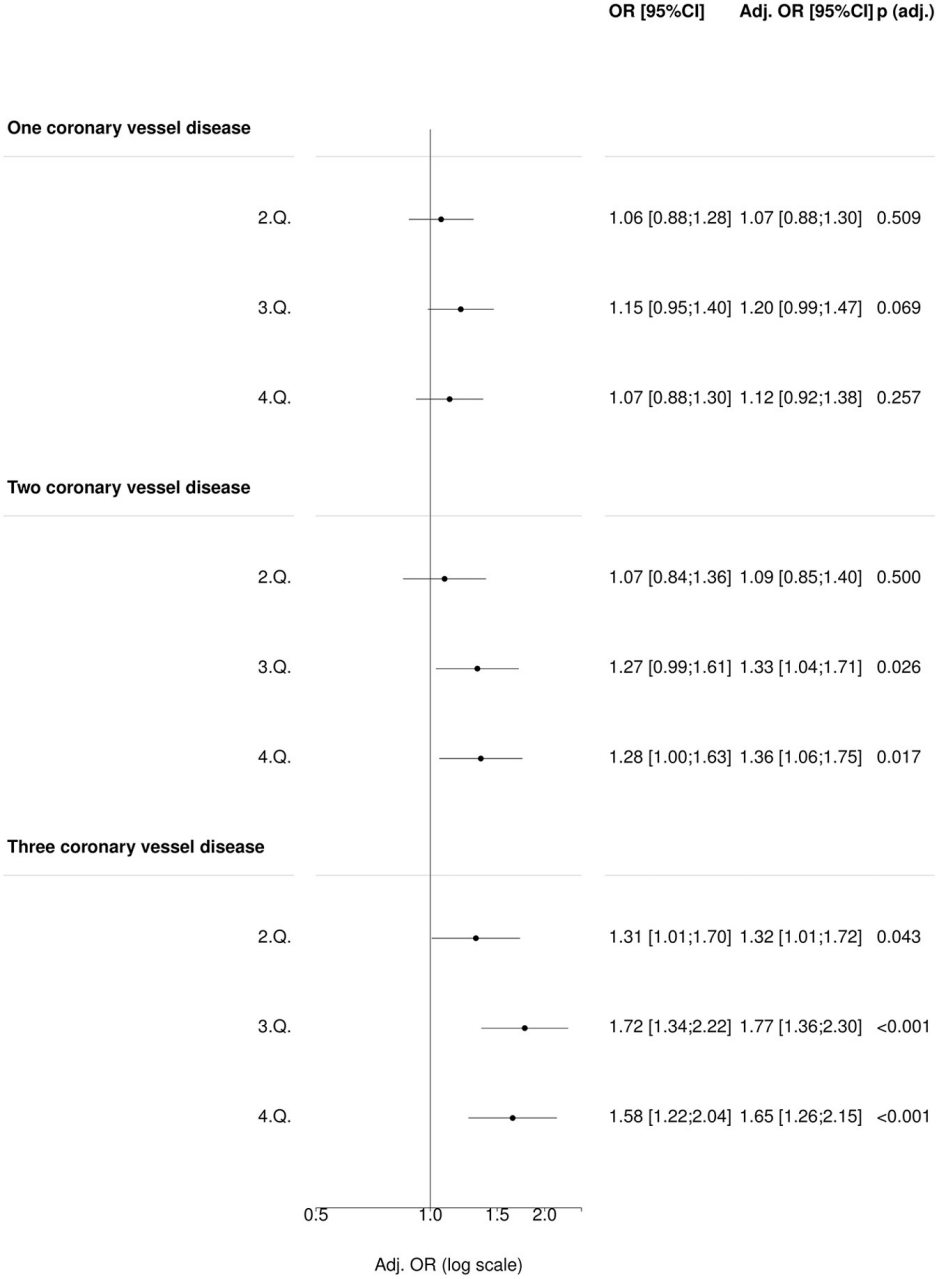
Association of blood pressure uGRS with CHD (multilevel outcome) in COGEN cohort. (N = 4,809).

Among patients referred for CAG, no association between uGRS quartiles and one vessel coronary heart disease was identified neither in the unadjusted or the adjusted models (as compared with no significant obstructive vessel disease, Fig 2).

Among patients with two coronary vessel disease the multivariable analysis showed an association between 3^rd^ and 4^th^ uGRS quartiles and increased odds of significant coronary vessel disease with odds ratios OR 1.33 and 1.36 (95% CI 1.04–1.71, p = 0.03 and 95% CI 1.06–1.75, p = 0.02 respectively, Fig 2).

Among patients with three coronary vessel disease, both the unadjusted and adjusted analyses showed that patients in 2^nd^, 3^rd^ and 4^th^ uGRS quartiles had increased odds of significant coronary disease when compared to patients in 1^st^ quartile (OR 1.32, 95% CI 1.01–1.72; OR 1.77 95% CI 1.36–2.30 and OR 1.65 95% CI 1.26–2.15 respectively, Fig 2).

Similar results were observed using wGRS (S5 Fig).

### Population based analyses (Inter99)

Risk of prevalent MI associated with hypertension GRS was evaluated in a population based setting using the Inter99 cohort. Baseline demographics are shown in S5 Table. Here we found that 4^th^ quartile of hypertension associated GRS was significantly associated with increased risk of developing myocardial infarction in unadjusted and adjusted models with 1.98 and 2.02 fold increased risk of MI respectively (95% CI 1.08–3.64 and 1.09–3.75) (S6 Fig).

We also evaluated the pleiotropy of hypertension uGRS with diabetes, smoking, BMI and hyperlipidemia. Our results showed no evidence for pleiotropy between hypertension uGRS and the above mentioned CHD risk factors (S6 Table).

### Sensitivity analyses (COGEN and Inter99)

**Fig 3:**
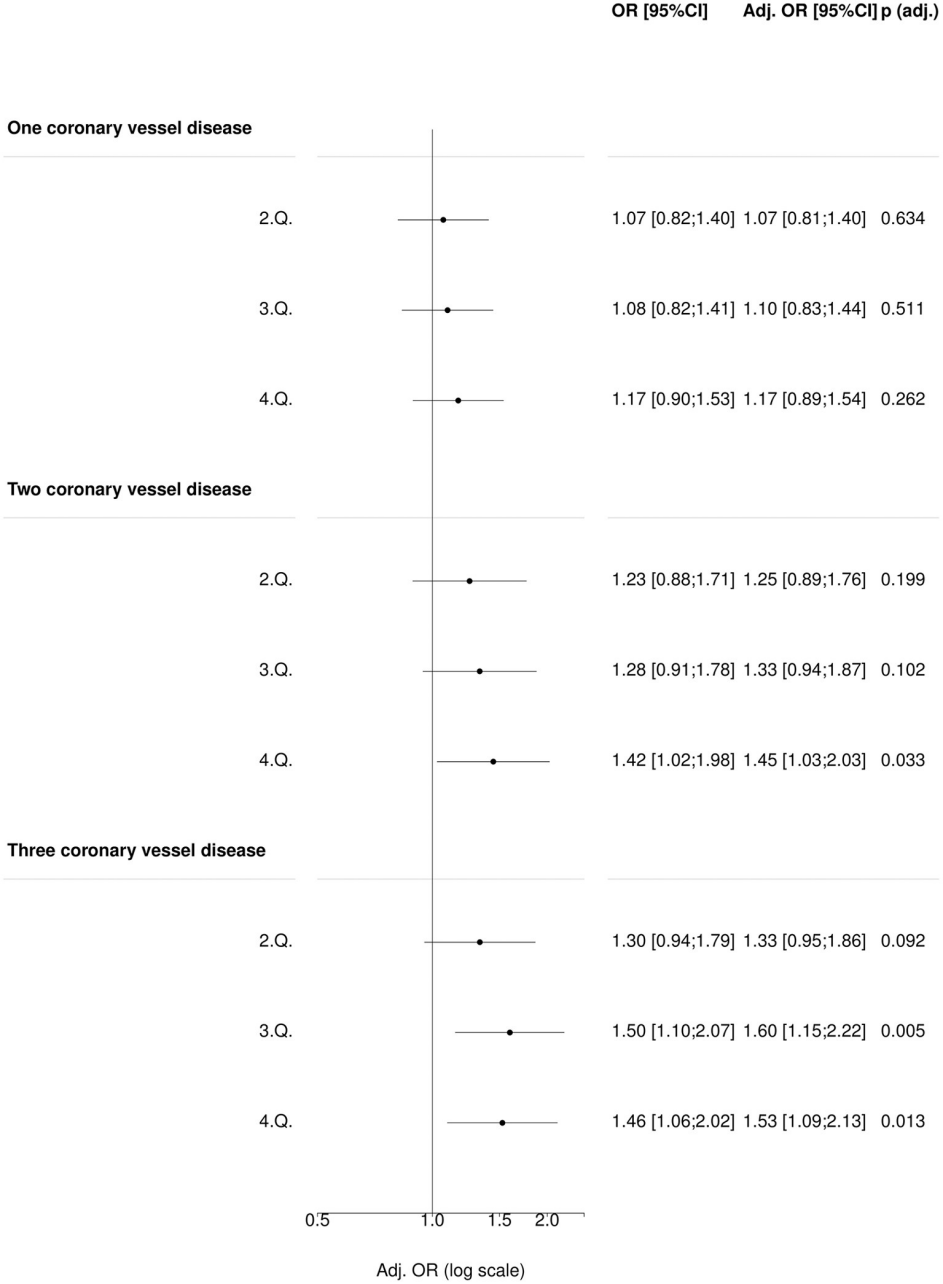
Association of blood pressure uGRS with CHD in individuals with no pre-hypertension (COGEN) (N = 2,707).

We performed three sensitivity analyses. First, we evaluated whether hypertension GRS was associated with CHD and burden of disease in individuals not diagnosed with hypertension in both COGEN and Inter99 populations. In COGEN, we generally observed the same effect sizes as in the main analysis, however the association of 3^rd^ uGRS quantile with 2-coronary vessel disease did not remain significant (p = 0.10, Fig 3).

**Fig 4:**
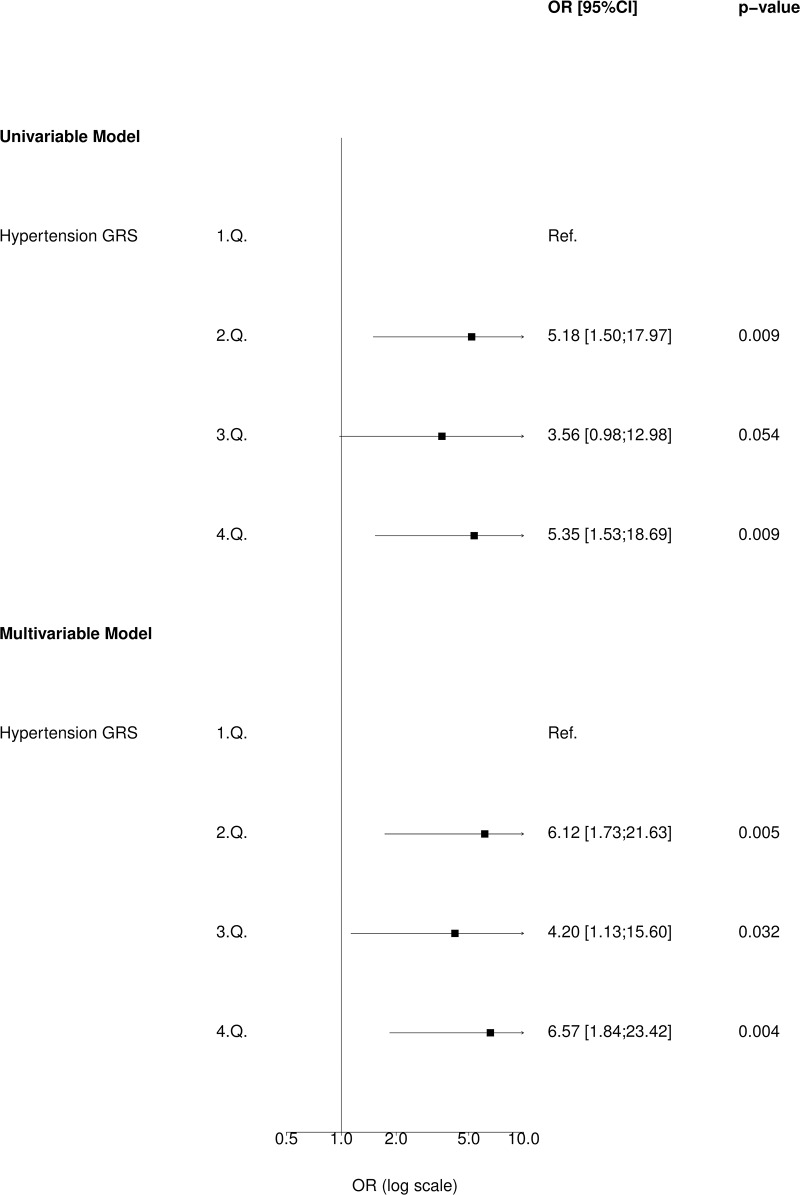
Association of blood pressure uGRS with AMI in individuals with no pre-hypertension (Inter99) (N = 3,742).

In Inter99, we observed higher ORs and larger CIs (Fig 4) compared to the main analysis. Furthermore, 2^nd^ uGRS quantile was statistically significant in this stratified analysis both in the unadjusted and adjusted model (OR 5.18; 95% CI 1.50–17.97; p<0.01 and OR 6.12; 95% CI 1.73–21.63; p<0.01, Fig 4). The same observation was made regarding 3^rd^ uGRS quantile (OR 3.56; 95% CI 0.98–12.98; p = 0.05 and OR 4.20; 95% CI 1.13–15.60; p = 0.03, Fig 4).

Second, we ascertained to which extent each of the three blood pressure traits, namely SBP, DBP and PP, contributed to the risk of CHD in COGEN population. The adjusted model with significant CHD entered as the outcome variable showed that 4^th^ unweighted systolic blood pressure (uSBP) GRS quantile was associated with increased CHD risk (S7 Fig). Unweighted pulse pressure (uPP) GRS’ 2^nd^, 3^rd^ and 4^th^ quartiles were significantly associated with increased odds of CHD (S9 Fig). No association with increased odds of CHD was observed in relation to unweighted diastolic blood pressure (uDBP) GRS (S8 Fig).

Third, we performed multinomial logistic regression analysis where individuals with previous history of acute myocardial infarction (AMI) were excluded. The reason was to verify whether these individuals carry a higher risk of developing CHD. We observed no differences in estimates compared to the main analysis (S10 Fig).

## Discussion

This hypertension GRS study involving 4,809 patients who were admitted to a coronary angiography (COGEN) and a population based cohort (Inter99) had six essential findings. First, we observed an association between higher hypertension GRS with greater risk of coronary heart disease. Second, our study demonstrated a dose-response pattern between the hypertension GRS and the severity of coronary heart disease among patients referred to coronary angiography. Third, the study demonstrated that, a hypertension GRS was independently associated with increased odds of developing CHD in a population based setting. Fourth, the association of hypertension GRS with increased odds of CHD was regardless of being diagnosed with hypertension or receiving antihypertensive treatment. Fifth, PP and SBP susceptibility GRS were the strongest predictors of coronary artery disease development. Sixth, there was no evidence of pleiotropy between hypertension GRS and diabetes, smoking, BMI or hyperlipidemia.

Our findings add to the notion of hypertension being strongly associated with risk of developing CHD. However, we were also able to demonstrate that patients with a stronger genetic predisposition to develop hypertension (based on known hypertension SNPs) suffered from more severe coronary heart disease compared to those with less genetic burden to develop hypertension. These findings are of particular interest, given that the SNPs included in the present study account for only ~2% of the overall heritability of hypertension and are correlated with risk of CHD development, but also the degree of disease [19]. Thus, with a better understanding and identification the genetic architecture predisposing patients to develop hypertension, more of the disease variability can be accounted for, which translates into an improved risk prediction of CHD development and severity. Yet, the association of different SNPs with increased risk of hypertension does not give any insight into the mechanism of development of hypertension. SNPs can affect molecular function and contribute to disease risk through different mechanisms such as altered transportations rate of a protein, altered protein folding or message processing and message stability. We also showed that individuals had an increased risk of hypertension regardless of being diagnosed and/or treated for hypertension. An argument for this finding can be great arterial stiffness in the non-hypertensive individuals which represents an early stage in the pathogenesis of hypertension. In 1,564 non-hypertensive Framingham Heart Study third-generation cohort participants, Andersson et al. [20] showed that non-hypertensive adults had higher vascular stiffness if they were offspring of one or two parents with hypertension compared to those with no familial predisposition. Unfortunately, due to great missingness of self-reported information on familial hypertension we could not account for this major confounder.

Generally, our findings are in agreement with other recent studies. Hoffmann et al. [3] investigated the association of SBP, DBP and PP susceptibility GRSs with MI/CHD and the authors found that a GRS constructed from 70 SNPs were highly significantly associated with MI/CHD with odds of 1.06 (p = 1.1 x 10^−45^) per 1 mmHg increase in SNP-based BP. Likewise, Surendran et. al. [19] found that blood pressure traits such as SBP, DBP and PP were positively associated with increased CHD risk. The results of our study are not suggestive of an association between DBP susceptibility GRS and increased odds of CHD neither as binary nor as multilevel outcome. Frankling et al [21] showed in 6,539 Framingham Heart Study participants, who were not on antihypertensive therapy at baseline, that there was a gradual shift from DBP to SBP and then to PP as predictors of CHD risk with increasing age. The same was concluded by Franklin et al. in 2013 [22] when discussing the most important contributions of the Framingham Heart Study on epidemiology of blood pressure. Our study included approximately 20% individuals under the age of 50 years, which could explain the lack of association between the DBP susceptibility GRS and increased CHD risk. Another study looked at the impact of cumulative hypertension GRS on coronary heart disease [4]. The study showed that the GRS was associated with increased risk of CHD when comparing upper and lower quintiles of the GRS (OR 1.38, p = 4.3×10^−23^). This is consistent with our findings, where we found 32% and 27% higher odds of prevalent CHD among patients in the 3^rd^ and 4^th^ quartiles compared to those in the lowest quartile.

To our knowledge, the present study is the first to demonstrate a dose-response pattern associated with the genetic burden of hypertension associated SNPs and severity of CHD. Notably, we found an association of 3^rd^ and 4^th^ uGRS quantiles with increased odds of developing two- and three coronary vessel disease in patients referred to angiography compared to 1^st^ uGRS quartile.

Coronary heart disease risk prediction has a central role in daily clinical practice and strong efforts are made to improve risk assessment methodology, as traditional risk factors do not identify most individuals with high CHD risk. Recently, increased attention has been directed towards the identification of genetic risk markers and quantification of the improvement in risk prediction models that include GRS in general [23]. As far as our knowledge, only one study, directly investigated the effect of inclusion of hypertension GRS in a cardiovascular risk stratification score system. The authors found that inclusion of hypertension GRS of the aggregated risk effects of the associated loci into a cardiovascular risk prediction model could improve both patient risk classification and blood pressure regulation [24]. Several studies found positive association between GRS awareness on “soft” end points (e.g. information seeking on the disease) but only few succeeded to find associations in “hard” endpoints (e.g. mortality). For example, one study showed that individuals which were aware of their personal CHD risk based on CHD susceptibility GRS seemed to show an increased tendency to seek and share disease related information [25]. Khera et al. [26] investigated to which extent increased CHD genetic risk can be offset by a healthy lifestyle. The authors found that individuals in the top quintile of the CHD susceptibility GRS had a substantially lower risk of coronary events if they adhered to a favorable lifestyle [26].

As we observed, information on the effect of including GRSs in cardiovascular risk prediction score systems are sparse, especially when referring to hypertension GRS. However, the available studies suggest that awareness of genetic susceptibility improves regulation of some of the risk factors included in the traditional prediction models (e.g. hypertension and lifestyle). Future studies investigating whether awareness of hypertension GRS decreases blood pressure and increases patient compliance to antihypertensive therapy are needed. Moreover, studies investigating performance of CHD risk prediction models when hypertension GRS is included are warranted in order to identify as many patients at risk as possible. Furthermore, a more sensitive and specific cardiovascular risk prediction score system could be beneficial especially when referring to individuals with increased risk of CHD involving multiple coronary vessels as this patient group has increased risk of developing MI induced comorbidity such as heart failure or kidney disease [27], [28].

### Study strengths and limitations

This study’s major strength is represented by inclusion of a rather large and clinically representative cohort of individuals referred to coronary angiography—including information in the extent of CHD.

The limitations are largely related to the observational nature of the study. Furthermore, our population is highly selected, as its main inclusion criteria is admission to coronary angiography, mainly due to symptoms or clinical presentation where CHD is suspected. However, by including a population based cohort (Inter99) which yielded similar findings in terms of a significant association between hypertension GRS and MI adds to the external validity of the study. Yet, the results using data of the Inter99 study might be underestimated due to the low follow-up rate (66%).

Our population may not extend to other non-Europeans Caucasian populations, which can lead to difficulty in reproducing these results worldwide.

## Perspectives

A hypertension GRS comprised of a large number of GWAS identified SNPs was associated with increased burden of CHD. This finding is an important step in building and evaluating absolute risk models for the general population. Early and accurate identification of individuals at risk might implicitly lead to more effective implementation of preventative lifestyle modifications, improve treatment compliance and intensive follow-up.

## Conclusion

In the present study, we identified a significant dose-response pattern between a GRS on GWAS identified common genetic variants associated with systolic blood pressure, diastolic blood pressure, or pulse pressure and the risk of hypertension, CHD and also degree of coronary vessel disease such as multi vessel disease in individuals who were admitted to a coronary angiography. The association of hypertension GRS with increased odds of CHD was independent of individuals being diagnosed or receiving treatment for hypertension. PP and SBP GRS seemed to be the strongest determinants of CHD when compared with DBP GRS. We showed no evidence for pleiotropy between hypertension GRS and diabetes, smoking, BMI or hyperlipidemia in a population-based sample. Whether the inclusion of hypertension GRS in known CHD risk models will improve survival remains to be determined.

## Supporting information

S1 File Genotyping, imputation and quality control procedures. (DOCX)

S1 Table List of SNP’s from literature (Hoffmann et.al) excluded due to minor allele count <20. (DOCX)

S2 Table List of SNPs from literature (Hoffmann et. al) with no proxies captured in the population. (DOCX)

S3 Table List of SNPs associated with blood pressure at genome-wide significance used to calculate cumulated weighted genetic risk score. (DOCX)

S4 Table Results of ANOVA and generalized linear model (GLM) analysis for difference in hypertension genetic risk score according to number of affected coronary vessels. (DOCX)

S5 Table Demographics Inter99 (N = 4,912). (DOCX)

S6 Table Evaluation of pleiotropy between hypertension GRS and BMI, hyperlipidemia, smoking and diabetes (Inter99) (N = 4,192). (DOCX)

S1 Fig Distribution of weighted and unweighted hypertension genetic risk score according to prevalent hypertension (N = 4,809). (TIFF)

S2 Fig Distribution of weighted and unweighted hypertension genetic risk score according to number of affected coronary vessels (N = 4,809). (TIFF)

S3 Fig Association of blood pressure unweighted genetic risk score with prevalent hypertension (binary outcome) in COGEN cohort (N = 4,809). (TIFF)

S4 Fig Association of blood pressure weighted genetic risk score with coronary heart disease (binary outcome) in COGEN cohort (N = 4,809). (TIFF)

S5 Fig Association of blood pressure weighted genetic risk score with coronary heart disease (multilevel outcome) in COGEN cohort (N = 4,809). (TIFF)

S6 Fig Association of blood pressure unweighted genetic risk score with prevalent acute myocardial infarction (binary outcome) in Inter99 population (N = 4,912). (TIFF)

S7 Fig Association of systolic blood pressure unweighted genetic risk score with coronary heart disease (binary outcome) in COGEN cohort (N = 4,809). (TIFF)

S8 Fig Association of diastolic blood pressure unweighted genetic risk score with coronary heart disease (binary outcome) in COGEN cohort (N = 4,809). (TIFF)

S9 Fig Association of pulse pressure genetic risk score with coronary heart disease (binary outcome) in COGEN cohort. (TIFF)

S10 Fig Association of blood pressure unweighted genetic risk score with coronary heart disease (multilevel outcome) in COGEN cohort where individuals with previous history with acute myocardial infarction were excluded (N = 4,109). (TIFF)
