# Correction: Hypertension genetic risk score is associated with burden of coronary heart disease among patients referred for coronary angiography

**DOI:** 10.1371/journal.pone.0293765

**Published:** 2023-10-26

**Authors:** Maria Lukács Krogager, Regitze Kuhr Skals, Emil Vincent R. Appel, Theresia M. Schnurr, Line Engelbrechtsen, Christian Theil Have, Oluf Pedersen, Thomas Engstrøm, Dan M. Roden, Gunnar Gislason, Henrik Enghusen Poulsen, Lars Køber, Steen Stender, Torben Hansen, Niels Grarup, Charlotte Andersson, Christian Torp-Pedersen, Peter E. Weeke

In the Ethics subsection of the Methods, there is an error in the paragraph. The correct paragraph is: All data were de-identified prior to analyses. Written informed consent was obtained from all participants included in the Inter99 study. The ethics committee of Region North Jutland (N-20140048) approved the project and that blood samples from COGEN may be used without written informed consent from patients. COGEN also has permission from the Data Protection Agency (00916 GEH-2010-001). The Inter99 study was approved by the Scientific Ethics Committee of the Capital Region of Denmark (KA98155) and registered as clinical trial (ClinicalTrials.gov; ID-no: NCT00289237). Both studies were performed in accordance with the principles of the Declaration of Helsinki.
