# Supplementary material for: Hypertension genetic risk score is associated with burden of coronary heart disease among patients referred for coronary angiography
Source: PLoS One. 2018 Dec 19;13(12):e0208645. doi: 10.1371/journal.pone.0208645 (PMC6300273; doi:10.1371/journal.pone.0208645)
Supplement: S1 File — (DOCX) [file pone.0208645.s001.docx]

**S1 File. Genotyping, Imputation and Quality control procedures**

In the Copenhagen Cardiovascular Genetic study (COGEN), DNA was genotyped in 5,904 participants using the Illumina HiScan platform at the Novo Nordisk Foundation Center for Basic Metabolic Research’s laboratory at Symbion, Copenhagen, Denmark. The standard pipeline included in Illumina Genome Studio software was used for the genotype calling. SNPs were mapped to GRCh37.p13 assembly (i.e. hg19). Before running quality control (QC) analyses, a total of 5,904 individuals and 547,644 SNPs were included. After QC, a total of 5,128 individuals and 539,004 SNPs were eligible for further investigation. Prior to genotype imputation, SNPs and/or individuals were excluded using following criteria:

1) a genotype call-rate below 95% (removed n=46 individuals),

2) extreme positive or negative inbreeding coefficients (removed n=56 individuals),

3) individuals of divergent ancestry using Principal Component Analysis (PCA) (removed n=566 individuals),

4) first degree relative relations found by Identical By Descent (IBD) analysis where only the relative with the highest call-rate for each pedigree-pair was retained (removed n=63 individuals),

5) duplicated individuals (excluded n=16 individuals),

6) sex discordant information between genotype and phenotype data (removed n=29 individuals).

QC analyses were performed using three different softwares: Python, R and PLINK.

Before imputation, genotypes from the Illumina CoreExome Chip were aligned to the forward strand of GRCh37 using data stored in a VCF, according to the instructions provided by the Sanger imputation service. Imputation was performed on data including individuals with other ancestry than Danish. After imputation we excluded non-autosomal variants, variants with a more than 5% missing calls, variants which had a minor allele frequency of less than 0.05, or which were not in Hardy-Weinberg equibrilium (p-value of at most 10^e-4^), leaving in total 260,792 variants genotyped in 5,671 individuals. Genotypes were prephased using Eagle and imputed using pbwt to the Haplotype Reference Consortium (r1.1) via the Sanger imputation server on January 5, 2017.
