# Supplementary material for: Hypertension genetic risk score is associated with burden of coronary heart disease among patients referred for coronary angiography
Source: PLoS One. 2018 Dec 19;13(12):e0208645. doi: 10.1371/journal.pone.0208645 (PMC6300273; doi:10.1371/journal.pone.0208645)
Supplement: S1 Table — (DOCX) [file pone.0208645.s002.docx]

**S1 Table. List of SNP’s from literature (Hoffmann et.al) excluded due to minor allele count <20**

| **Trait** | **SNP** | **Chr^a^** | **Pos^b^** | **Effect allele** | **Other allele** | **Reported alleles** | **EAF^c^** | **Effect** | **Units** | **P** | **N** |
| --- | --- | --- | --- | --- | --- | --- | --- | --- | --- | --- | --- |
| DBP | rs187222839 | 11 | 97944152 | T | C | T/C | 0.997 | -2.186 | mmHg | 9.30E-09 | 321262 |
| DBP | rs183335240 | 18 | 59096824 | A | G | A/G | 0.998 | 2.305 | mmHg | 1.30E-08 | 321262 |
| SBP | rs528266117 | 3 | 52729780 | C | T | C/CAATT | 0.999 | -24.445 | mmHg | 7.40E-09 | 321262 |
| PP | rs528266117 | 3 | 52729780 | C | T | C/CAATT | 0.999 | -15.799 | mmHg | 1.40E-08 | 321262 |
| SBP | rs185695143 | 5 | 10860486 | C | T | C/T | 1 | -20.232 | mmHg | 2.50E-08 | 321262 |
| PP | rs530280439 | 12 | 127031062 | C | T | C/T | 1 | -17.469 | mmHg | 4.80E-09 | 321262 |
| PP | [rs538839447](http://www.ncbi.nlm.nih.gov/SNP/snp_ref.cgi?rs=538839447) | 4 | 47378386 | G | A | G/A | 1 | -14.405 | mmHg | 1.40E-08 | 321262 |
| DBP | rs369386096 | 20 | 23502129 | C | G | C/G | 1 | -9.788 | mmHg | 3.60E-08 | 321262 |
| DBP | [rs767679894](http://www.ncbi.nlm.nih.gov/SNP/snp_ref.cgi?rs=767679894) | 1 | 8472788 | T | A | T/A | 1 | -7.916 | mmHg | 1.90E-09 | 321262 |
| PP | rs114053299 | 5 | 12780703 | A | G | A/G | 1 | -6.487 | mmHg | 4.80E-08 | 321262 |
| PP | rs184145372 | 4 | 159150358 | T | C | T/C | 1 | -5.776 | mmHg | 1.80E-08 | 321262 |
| SBP | rs115381894 | 11 | 109019018 | G | A | G/A | 1 | -5.212 | mmHg | 3.70E-08 | 321262 |

^a^Chr=chromosome

^b^Pos=Position

^c^EAF= Effect allele frequency
