# Supplementary material for: Hypertension genetic risk score is associated with burden of coronary heart disease among patients referred for coronary angiography
Source: PLoS One. 2018 Dec 19;13(12):e0208645. doi: 10.1371/journal.pone.0208645 (PMC6300273; doi:10.1371/journal.pone.0208645)
Supplement: S2 Table — (DOCX) [file pone.0208645.s003.docx]

**S2 Table. List of SNPs from literature (Hoffmann et. al) with no proxies captured in the population**

| **Trait** | **SNP** | **Chr^a^** | **Pos^b^** | **Effect allele** | **Other allele** | **Reported alleles** | **EAF^c^** | **Effect** | **Units** | **P** | **N** |
| --- | --- | --- | --- | --- | --- | --- | --- | --- | --- | --- | --- |
| PP | rs2914609 | 5 | 121287061 | T | C | T/C | 0.175 | 0.188 | mmHg | 9.40E-09 | 321262 |
| PP | rs1966323 | 7 | 116571847 | T | C | T/C | 0.31 | 0.166 | mmHg | 1.60E-09 | 321262 |
| PP | rs56143613 | 16 | 56328811 | A | G | A/G | 0.324 | 0.282 | mmHg | 1.50E-09 | 321262 |
| SBP | rs4788913 | 17 | 73950216 | G | A | G/A | 0.34 | 0.283 | mmHg | 1.80E-12 | 321262 |

| SBP | rs5794844 | 11 | 112960099 | G | G | G/G | 0.42 | -0.355 | mmHg | 3.50E-08 | 321262 |
| --- | --- | --- | --- | --- | --- | --- | --- | --- | --- | --- | --- |

| PP | rs5794844 | 11 | 112960099 | G | G | G/G | 0.42 | -0.268 | mmHg | 3.10E-09 | 321262 |
| --- | --- | --- | --- | --- | --- | --- | --- | --- | --- | --- | --- |
| DBP | rs7989823 | 13 | 110959643 | A | C | A/C | 0.434 | -0.15 | mmHg | 5.60E-09 | 321262 |
| PP | rs4140574 | 6 | 56099424 | T | A | T/A | 0.438 | 0.231 | mmHg | 4.30E-19 | 321262 |
| PP | rs2899463 | 15 | 50938978 | T | C | T/C | 0.488 | 0.153 | mmHg | 1.30E-09 | 321262 |
| PP | rs1027647 | 15 | 63374825 | C | A | C/A | 0.515 | 0.153 | mmHg | 3.30E-09 | 321262 |
| DBP | rs567058829 | 1 | 175111760 | C | T | C/T | 0.536 | 0.197 | mmHg | 3.60E-08 | 321262 |
| PP | rs7226020 | 17 | 6473828 | T | C | T/C | 0.563 | -0.232 | mmHg | 2.10E-17 | 321262 |
| PP | rs367700296 | 14 | 98597422 | G | A | G/A | 0.635 | -0.274 | mmHg | 9.80E-09 | 321262 |
| PP | rs7519279 | 1 | 169207361 | G | C | G/C | 0.644 | 0.21 | mmHg | 1.10E-13 | 321262 |
| DBP | rs67833703 | 17 | 3888437 | C | T | C/T | 0.679 | -0.147 | mmHg | 1.10E-08 | 321262 |
| PP | rs4980877 | 12 | 418916 | C | T | C/T | 0.716 | -0.162 | mmHg | 2.70E-08 | 321262 |
| DBP | rs34594435 | 7 | 72977249 | C | T | C/T | 0.804 | 0.183 | mmHg | 1.30E-09 | 321262 |
| PP | rs200688233 | 19 | 10372360 | G | C | G/C | 0.807 | -0.314 | mmHg | 3.90E-08 | 321262 |
| DBP | rs507666 | 9 | 136149399 | G | A | G/A | 0.815 | 0.169 | mmHg | 1.70E-08 | 321262 |
| PP | rs57448815 | 21 | 30123533 | A | G | A/G | 0.86 | -0.227 | mmHg | 6.00E-09 | 321262 |
| DBP | rs76627715 | 7 | 80387316 | T | C | T/C | 0.876 | 0.216 | mmHg | 1.10E-09 | 321262 |
| SBP | rs80073370 | 8 | 19833156 | A | T | A/T | 0.918 | 0.404 | mmHg | 8.40E-09 | 321262 |
| DBP | rs117638970 | 15 | 69675605 | C | T | C/T | 0.975 | 0.484 | mmHg | 2.20E-08 | 321262 |
| DBP | rs139491786 | 16 | 2086421 | C | T | C/T | 0.993 | 1.155 | mmHg | 1.20E-09 | 321262 |

^a^Chr=chromosome

^b^Pos=Position

^c^EAF= Effect allele frequency
