# Supplementary material for: Hypertension genetic risk score is associated with burden of coronary heart disease among patients referred for coronary angiography
Source: PLoS One. 2018 Dec 19;13(12):e0208645. doi: 10.1371/journal.pone.0208645 (PMC6300273; doi:10.1371/journal.pone.0208645)
Supplement: S3 Table — (DOCX) [file pone.0208645.s004.docx]

**S3 Table. List of SNPs associated with blood pressure at genome-wide significance used to calculate cumulated weighted genetic risk score**

| GRS type | Trait | SNP | Chr | Pos | Effect allele | Other allele | Reported alleles | EAF | Effect | Units | P | N |
| --- | --- | --- | --- | --- | --- | --- | --- | --- | --- | --- | --- | --- |
| BP | DBP | rs10751962 | 10 | 4172711 | C | T | C/T | 0.087 | -0.219 | mmHg | 4.60E-08 | 321262 |
| BP | PP | rs10418305 | 19 | 15278808 | C | G | C/G | 0.096 | -0.33 | mmHg | 3.50E-16 | 321262 |
| BP | DBP | rs7116797 | 11 | 116707338 | A | G | A/G | 0.108 | 0.214 | mmHg | 1.60E-09 | 321262 |
| BP | SBP | rs4551692 | 10 | 102556453 | G | A | G/A | 0.112 | -0.419 | mmHg | 1.60E-11 | 321262 |
| BP | DBP | rs4551692 | 10 | 102556453 | G | A | G/A | 0.112 | -0.255 | mmHg | 1.30E-11 | 321262 |
| BP | PP | rs516143 | 6 | 159696185 | G | C | G/C | 0.141 | 0.314 | mmHg | 1.40E-16 | 321262 |
| BP | DBP | rs61879810 | 11 | 31821467 | A | G | A/G | 0.154 | 0.198 | mmHg | 1.20E-09 | 321262 |
| BP | DBP | rs1761870 | 6 | 117264985 | G | A | G/A | 0.171 | 0.175 | mmHg | 3.50E-09 | 321262 |
| BP | PP | rs2914609 | 5 | 121287061 | T | C | T/C | 0.175 | 0.188 | mmHg | 9.40E-09 | 321262 |
| BP | PP | rs2706110 | 2 | 178092162 | T | C | T/C | 0.189 | 0.182 | mmHg | 9.30E-09 | 321262 |
| BP | SBP | rs200541 | 16 | 24733141 | A | G | A/G | 0.194 | -0.281 | mmHg | 8.50E-09 | 321262 |
| BP | PP | rs8103992 | 19 | 19665643 | A | C | A/C | 0.201 | 0.198 | mmHg | 9.50E-10 | 321262 |
| BP | SBP | rs4110517 | 10 | 96650328 | A | G | A/G | 0.21 | -0.282 | mmHg | 1.10E-09 | 321262 |
| BP | PP | rs2289125 | 11 | 89224453 | A | C | A/C | 0.213 | -0.347 | mmHg | 1.50E-30 | 321262 |
| BP | SBP | rs2289125 | 11 | 89224453 | A | C | A/C | 0.213 | -0.274 | mmHg | 1.60E-09 | 321262 |
| BP | PP | rs1322639 | 6 | 169587103 | G | A | G/A | 0.22 | -0.302 | mmHg | 1.20E-23 | 321262 |
| BP | SBP | rs13112725 | 4 | 106911742 | G | C | G/C | 0.241 | -0.297 | mmHg | 2.10E-11 | 321262 |
| BP | DBP | rs13112725 | 4 | 106911742 | G | C | G/C | 0.241 | -0.15 | mmHg | 3.20E-08 | 321262 |
| BP | PP | rs813412 | 10 | 32284825 | C | T | C/T | 0.246 | 0.178 | mmHg | 2.50E-09 | 321262 |
| BP | SBP | rs3011549 | 13 | 113634937 | A | C | A/C | 0.253 | 0.326 | mmHg | 1.20E-11 | 321262 |
| BP | PP | rs10838433 | 11 | 45233473 | G | A | G/A | 0.261 | -0.195 | mmHg | 5.30E-12 | 321262 |
| BP | DBP | rs6957161 | 7 | 131361319 | A | G | A/G | 0.262 | 0.157 | mmHg | 1.70E-09 | 321262 |
| BP | SBP | rs6957161 | 7 | 131361319 | A | G | A/G | 0.262 | 0.256 | mmHg | 2.10E-09 | 321262 |
| BP | PP | rs1250247 | 2 | 216299629 | C | G | C/G | 0.271 | 0.279 | mmHg | 1.10E-21 | 321262 |
| BP | SBP | rs1250247 | 2 | 216299629 | C | G | C/G | 0.271 | 0.31 | mmHg | 1.10E-12 | 321262 |
| BP | DBP | rs35654783 | 15 | 44018656 | T | C | T/C | 0.277 | 0.166 | mmHg | 1.30E-10 | 321262 |
| BP | SBP | rs7161323 | 14 | 53366149 | C | T | C/T | 0.286 | -0.275 | mmHg | 5.70E-11 | 321262 |
| BP | DBP | rs7161323 | 14 | 53366149 | C | T | C/T | 0.286 | -0.147 | mmHg | 1.20E-08 | 321262 |
| BP | DBP | rs7928655 | 11 | 13300252 | C | G | C/G | 0.297 | -0.143 | mmHg | 1.60E-08 | 321262 |
| BP | DBP | rs1876487 | 2 | 73114352 | A | C | A/C | 0.299 | -0.162 | mmHg | 1.50E-09 | 321262 |
| BP | PP | rs1966323 | 7 | 116571847 | T | C | T/C | 0.31 | 0.166 | mmHg | 1.60E-09 | 321262 |
| BP | PP | rs7856420 | 9 | 123839157 | G | C | G/C | 0.313 | 0.159 | mmHg | 5.50E-09 | 321262 |
| BP | PP | rs6434404 | 2 | 191494411 | A | G | A/G | 0.322 | 0.185 | mmHg | 1.30E-10 | 321262 |
| BP | SBP | rs6434404 | 2 | 191494411 | A | G | A/G | 0.322 | 0.311 | mmHg | 4.90E-13 | 321262 |
| BP | PP | rs56143613 | 16 | 56328811 | A | G | A/G | 0.324 | 0.282 | mmHg | 1.50E-09 | 321262 |
| BP | DBP | rs4984497 | 15 | 96635899 | T | C | T/C | 0.327 | 0.148 | mmHg | 5.40E-09 | 321262 |
| BP | DBP | rs1015538 | 7 | 99626035 | A | G | A/G | 0.33 | 0.137 | mmHg | 3.40E-08 | 321262 |
| BP | DBP | rs12630213 | 3 | 14954411 | C | T | C/T | 0.332 | 0.153 | mmHg | 4.80E-10 | 321262 |
| BP | SBP | rs12630213 | 3 | 14954411 | C | T | C/T | 0.332 | 0.276 | mmHg | 6.30E-12 | 321262 |
| BP | DBP | rs11631778 | 15 | 71606380 | G | A | G/A | 0.334 | -0.141 | mmHg | 1.10E-08 | 321262 |
| BP | SBP | rs4788913 | 17 | 73950216 | G | A | G/A | 0.34 | -0.283 | mmHg | 1.80E-12 | 321262 |
| BP | PP | rs4788913 | 17 | 73950216 | G | A | G/A | 0.34 | -0.154 | mmHg | 8.40E-09 | 321262 |
| BP | SBP | rs7225219 | 17 | 62407559 | T | A | T/A | 0.343 | -0.248 | mmHg | 2.50E-09 | 321262 |
| BP | PP | rs1475130 | 14 | 100225144 | T | C | T/C | 0.347 | -0.168 | mmHg | 6.90E-10 | 321262 |
| BP | PP | rs7714219 | 5 | 71654855 | G | C | G/C | 0.35 | -0.164 | mmHg | 3.80E-09 | 321262 |
| BP | PP | rs12050260 | 14 | 23761094 | T | C | T/C | 0.352 | 0.193 | mmHg | 1.60E-12 | 321262 |
| BP | SBP | rs685149 | 11 | 57657413 | A | G | A/G | 0.356 | -0.311 | mmHg | 1.50E-13 | 321262 |
| BP | PP | rs685149 | 11 | 57657413 | A | G | A/G | 0.356 | -0.186 | mmHg | 9.70E-12 | 321262 |
| BP | DBP | rs168643 | 5 | 50935900 | T | C | T/C | 0.356 | 0.139 | mmHg | 1.50E-08 | 321262 |
| BP | DBP | rs210156 | 6 | 33517362 | A | G | A/G | 0.359 | -0.148 | mmHg | 2.00E-09 | 321262 |
| BP | DBP | rs258494 | 5 | 75038718 | C | G | C/G | 0.367 | 0.216 | mmHg | 4.50E-19 | 321262 |
| BP | DBP | rs10747570 | 12 | 50509937 | A | G | A/G | 0.371 | 0.184 | mmHg | 4.30E-14 | 321262 |
| BP | SBP | rs10747570 | 12 | 50509937 | A | G | A/G | 0.371 | 0.256 | mmHg | 1.40E-10 | 321262 |
| BP | PP | rs4803457 | 19 | 41861359 | T | C | T/C | 0.372 | 0.149 | mmHg | 1.50E-08 | 321262 |
| BP | PP | rs7586597 | 2 | 9300092 | G | T | G/T | 0.373 | -0.154 | mmHg | 7.00E-09 | 321262 |
| BP | DBP | rs4295 | 17 | 61556298 | C | G | C/G | 0.376 | 0.14 | mmHg | 4.20E-08 | 321262 |
| BP | SBP | rs4295 | 17 | 61556298 | C | G | C/G | 0.376 | 0.24 | mmHg | 1.20E-08 | 321262 |
| BP | SBP | rs1694068 | 5 | 53283630 | T | A | T/A | 0.382 | -0.245 | mmHg | 7.00E-10 | 321262 |
| BP | PP | rs1694068 | 5 | 53283630 | T | A | T/A | 0.382 | -0.164 | mmHg | 6.40E-10 | 321262 |
| BP | PP | rs273957 | 7 | 137600690 | C | T | C/T | 0.385 | -0.147 | mmHg | 2.80E-08 | 321262 |
| BP | DBP | rs1063281 | 2 | 218668732 | C | T | C/T | 0.398 | 0.17 | mmHg | 9.50E-13 | 321262 |
| BP | SBP | rs1063281 | 2 | 218668732 | C | T | C/T | 0.398 | 0.247 | mmHg | 2.30E-10 | 321262 |
| BP | PP | rs7575523 | 2 | 59335104 | T | G | T/G | 0.399 | 0.142 | mmHg | 4.80E-08 | 321262 |
| BP | PP | rs114534 | 5 | 142533657 | G | A | G/A | 0.4 | 0.154 | mmHg | 2.40E-09 | 321262 |
| BP | SBP | rs35261357 | 16 | 75444572 | C | T | C/T | 0.402 | -0.265 | mmHg | 7.90E-12 | 321262 |
| BP | PP | rs35261357 | 16 | 75444572 | C | T | C/T | 0.402 | -0.216 | mmHg | 6.40E-17 | 321262 |
| BP | PP | rs786919 | 1 | 89281529 | A | G | A/G | 0.408 | 0.156 | mmHg | 1.50E-09 | 321262 |
| BP | SBP | rs786919 | 1 | 89281529 | A | G | A/G | 0.408 | 0.262 | mmHg | 1.30E-11 | 321262 |
| BP | SBP | rs7665304 | 4 | 109025379 | A | C | A/C | 0.419 | 0.22 | mmHg | 1.20E-08 | 321262 |
| BP | SBP | rs5794844 | 11 | 112960099 | G | G | GT/G | 0.42 | -0.355 | mmHg | 3.50E-08 | 321262 |
| BP | PP | rs5794844 | 11 | 112960099 | G | G | GT/G | 0.42 | -0.268 | mmHg | 3.10E-09 | 321262 |
| BP | PP | rs7019055 | 9 | 38088244 | A | G | A/G | 0.42 | 0.15 | mmHg | 7.10E-09 | 321262 |
| BP | PP | rs9303241 | 17 | 1978963 | T | A | T/A | 0.42 | 0.159 | mmHg | 1.00E-09 | 321262 |
| BP | PP | rs2393455 | 10 | 60374898 | C | A | C/A | 0.422 | -0.153 | mmHg | 4.90E-09 | 321262 |
| BP | SBP | rs360158 | 11 | 9753601 | G | A | G/A | 0.423 | -0.301 | mmHg | 1.40E-14 | 321262 |
| BP | DBP | rs360158 | 11 | 9753601 | G | A | G/A | 0.423 | -0.165 | mmHg | 4.70E-12 | 321262 |
| BP | PP | rs7605066 | 2 | 71529331 | C | T | C/T | 0.429 | 0.149 | mmHg | 6.30E-09 | 321262 |
| BP | SBP | rs12596053 | 16 | 4946794 | A | C | A/C | 0.431 | -0.275 | mmHg | 1.50E-12 | 321262 |
| BP | DBP | rs12596053 | 16 | 4946794 | A | C | A/C | 0.431 | -0.162 | mmHg | 9.60E-12 | 321262 |
| BP | DBP | rs7989823 | 13 | 110959643 | A | C | A/C | 0.434 | -0.15 | mmHg | 5.60E-09 | 321262 |
| BP | PP | rs4140574 | 6 | 56099424 | T | A | T/A | 0.438 | 0.231 | mmHg | 4.30E-19 | 321262 |
| BP | DBP | rs12405515 | 1 | 172357441 | G | T | G/T | 0.439 | 0.17 | mmHg | 9.70E-13 | 321262 |
| BP | PP | rs783621 | 1 | 42368035 | A | G | A/G | 0.442 | 0.207 | mmHg | 3.10E-16 | 321262 |
| BP | SBP | rs783621 | 1 | 42368035 | A | G | A/G | 0.442 | 0.289 | mmHg | 2.90E-14 | 321262 |
| BP | PP | rs7255 | 2 | 20878820 | T | C | T/C | 0.446 | -0.205 | mmHg | 2.10E-15 | 321262 |
| BP | SBP | rs7590201 | 2 | 227192443 | G | T | G/T | 0.449 | -0.216 | mmHg | 3.00E-08 | 321262 |
| BP | DBP | rs7590201 | 2 | 227192443 | G | T | G/T | 0.449 | -0.138 | mmHg | 7.60E-09 | 321262 |
| BP | PP | rs1350100 | 8 | 76054904 | A | G | A/G | 0.449 | 0.152 | mmHg | 4.20E-09 | 321262 |
| BP | PP | rs2761436 | 1 | 207919748 | C | T | C/T | 0.456 | -0.154 | mmHg | 2.10E-09 | 321262 |
| BP | DBP | rs62524579 | 8 | 144060955 | G | A | G/A | 0.466 | 0.147 | mmHg | 5.60E-10 | 321262 |
| BP | SBP | rs8073626 | 17 | 76790279 | C | T | C/T | 0.466 | 0.217 | mmHg | 1.40E-08 | 321262 |
| BP | SBP | rs62524579 | 8 | 144060955 | G | A | G/A | 0.466 | 0.244 | mmHg | 2.70E-10 | 321262 |
| BP | DBP | rs4653889 | 1 | 228112121 | A | G | A/G | 0.467 | 0.152 | mmHg | 1.20E-10 | 321262 |
| BP | PP | rs2049814 | 10 | 89787275 | A | G | A/G | 0.469 | -0.153 | mmHg | 1.80E-09 | 321262 |
| BP | SBP | rs56249585 | 16 | 65265702 | C | T | C/T | 0.47 | -0.373 | mmHg | 5.20E-09 | 321262 |
| BP | PP | rs56249585 | 16 | 65265702 | C | T | C/T | 0.47 | -0.337 | mmHg | 6.10E-14 | 321262 |
| BP | SBP | rs6090040 | 20 | 62692060 | A | C | A/C | 0.478 | 0.265 | mmHg | 1.40E-09 | 321262 |
| BP | SBP | rs10784502 | 12 | 66343810 | C | T | C/T | 0.487 | -0.235 | mmHg | 7.40E-10 | 321262 |
| BP | PP | rs10784502 | 12 | 66343810 | C | T | C/T | 0.487 | -0.188 | mmHg | 1.30E-13 | 321262 |
| BP | PP | rs2899463 | 15 | 50938978 | T | C | T/C | 0.488 | 0.153 | mmHg | 1.30E-09 | 321262 |
| BP | PP | rs13104866 | 4 | 38402183 | G | A | G/A | 0.489 | 0.151 | mmHg | 3.20E-09 | 321262 |
| BP | SBP | rs13104866 | 4 | 38402183 | G | A | G/A | 0.489 | 0.278 | mmHg | 3.80E-13 | 321262 |
| BP | PP | rs7666150 | 4 | 146814640 | T | C | T/C | 0.492 | 0.15 | mmHg | 5.60E-09 | 321262 |
| BP | DBP | rs7546498 | 1 | 1740255 | G | T | G/T | 0.493 | 0.147 | mmHg | 4.50E-10 | 321262 |
| BP | SBP | rs7546498 | 1 | 1740255 | G | T | G/T | 0.493 | 0.257 | mmHg | 2.80E-11 | 321262 |
| BP | SBP | rs7107356 | 11 | 47676170 | A | G | A/G | 0.494 | -0.309 | mmHg | 4.00E-16 | 321262 |
| BP | PP | rs34457140 | 17 | 1353920 | T | G | T/G | 0.494 | -0.163 | mmHg | 3.00E-10 | 321262 |
| BP | PP | rs7107356 | 11 | 47676170 | A | G | A/G | 0.494 | -0.159 | mmHg | 3.30E-10 | 321262 |
| BP | DBP | rs7107356 | 11 | 47676170 | A | G | A/G | 0.494 | -0.149 | mmHg | 1.70E-10 | 321262 |
| BP | PP | rs1333047 | 9 | 22124504 | A | T | A/T | 0.507 | -0.162 | mmHg | 2.00E-10 | 321262 |
| BP | DBP | rs13205180 | 6 | 51832494 | C | T | C/T | 0.507 | -0.143 | mmHg | 1.40E-09 | 321262 |
| BP | SBP | rs2050663 | 6 | 79753394 | T | C | T/C | 0.511 | -0.229 | mmHg | 3.60E-09 | 321262 |
| BP | DBP | rs2050663 | 6 | 79753394 | T | C | T/C | 0.511 | -0.174 | mmHg | 1.60E-13 | 321262 |
| BP | SBP | rs7951348 | 11 | 107081841 | C | T | C/T | 0.512 | -0.252 | mmHg | 3.60E-11 | 321262 |
| BP | PP | rs1027647 | 15 | 63374825 | C | A | C/A | 0.515 | 0.153 | mmHg | 3.30E-09 | 321262 |
| BP | PP | rs2440907 | 10 | 61638804 | G | T | G/T | 0.517 | -0.14 | mmHg | 4.40E-08 | 321262 |
| BP | PP | rs1923409 | 6 | 7728212 | G | A | G/A | 0.521 | -0.164 | mmHg | 1.50E-10 | 321262 |
| BP | SBP | rs2759308 | 15 | 81016227 | G | A | G/A | 0.523 | -0.276 | mmHg | 1.30E-12 | 321262 |
| BP | PP | rs2759308 | 15 | 81016227 | G | A | G/A | 0.523 | -0.145 | mmHg | 2.60E-08 | 321262 |
| BP | DBP | rs2759308 | 15 | 81016227 | G | A | G/A | 0.523 | -0.136 | mmHg | 9.00E-09 | 321262 |
| BP | SBP | rs66887589 | 4 | 120509279 | T | C | T/C | 0.525 | -0.214 | mmHg | 1.80E-08 | 321262 |
| BP | DBP | rs66887589 | 4 | 120509279 | T | C | T/C | 0.525 | -0.206 | mmHg | 9.20E-19 | 321262 |
| BP | DBP | rs6479908 | 10 | 65333648 | C | G | C/G | 0.526 | -0.142 | mmHg | 1.40E-09 | 321262 |
| BP | DBP | rs35565381 | 17 | 16175025 | T | C | T/C | 0.527 | -0.14 | mmHg | 3.30E-09 | 321262 |
| BP | DBP | rs460105 | 16 | 89682006 | T | C | T/C | 0.527 | 0.178 | mmHg | 3.60E-12 | 321262 |
| BP | PP | rs167479 | 19 | 11526765 | G | T | G/T | 0.528 | 0.179 | mmHg | 3.20E-08 | 321262 |
| BP | DBP | rs167479 | 19 | 11526765 | G | T | G/T | 0.528 | 0.251 | mmHg | 4.30E-22 | 321262 |
| BP | SBP | rs167479 | 19 | 11526765 | G | T | G/T | 0.528 | 0.41 | mmHg | 1.60E-21 | 321262 |
| BP | PP | rs869396 | 4 | 169688000 | C | A | C/A | 0.534 | 0.222 | mmHg | 5.90E-18 | 321262 |
| BP | DBP | rs3934939 | 13 | 114503990 | A | G | A/G | 0.535 | 0.163 | mmHg | 8.40E-09 | 321262 |
| BP | DBP | rs567058829 | 1 | 175111760 | C | T | C/CT | 0.536 | 0.197 | mmHg | 3.50E-08 | 321262 |
| BP | PP | rs10057188 | 5 | 77837789 | G | A | G/A | 0.538 | 0.153 | mmHg | 3.40E-09 | 321262 |
| BP | SBP | rs2360970 | 2 | 208409339 | G | C | G/C | 0.539 | -0.215 | mmHg | 1.80E-08 | 321262 |
| BP | SBP | rs6031435 | 20 | 42797358 | A | G | A/G | 0.54 | -0.214 | mmHg | 2.40E-08 | 321262 |
| BP | PP | rs6031435 | 20 | 42797358 | A | G | A/G | 0.54 | -0.17 | mmHg | 2.80E-11 | 321262 |
| BP | PP | rs62162674 | 2 | 85502236 | G | C | G/C | 0.544 | 0.181 | mmHg | 1.50E-12 | 321262 |
| BP | SBP | rs893929 | 4 | 144187380 | G | A | G/A | 0.545 | 0.233 | mmHg | 1.50E-09 | 321262 |
| BP | PP | rs1449544 | 8 | 76591880 | A | C | A/C | 0.546 | 0.2 | mmHg | 2.70E-15 | 321262 |
| BP | DBP | rs4475250 | 5 | 114375552 | G | A | G/A | 0.547 | 0.136 | mmHg | 7.10E-09 | 321262 |
| BP | SBP | rs4475250 | 5 | 114375552 | G | A | G/A | 0.547 | 0.243 | mmHg | 2.20E-10 | 321262 |
| BP | PP | rs10859580 | 12 | 94180616 | A | T | A/T | 0.548 | -0.143 | mmHg | 3.70E-08 | 321262 |
| BP | DBP | rs10198275 | 2 | 25130542 | A | C | A/C | 0.548 | 0.135 | mmHg | 5.90E-09 | 321262 |
| BP | SBP | rs4712656 | 6 | 22136262 | G | C | G/C | 0.554 | -0.227 | mmHg | 2.90E-09 | 321262 |
| BP | DBP | rs9882772 | 3 | 122110149 | T | C | T/C | 0.558 | -0.133 | mmHg | 1.50E-08 | 321262 |
| BP | DBP | rs6019378 | 20 | 47309716 | C | T | C/T | 0.559 | 0.156 | mmHg | 1.80E-11 | 321262 |
| BP | SBP | rs112925537 | 15 | 41334213 | A | T | A/AT | 0.56 | -0.413 | mmHg | 2.40E-10 | 321262 |
| BP | DBP | rs112925537 | 15 | 41334213 | A | T | A/AT | 0.56 | -0.204 | mmHg | 2.10E-08 | 321262 |
| BP | PP | rs1570350 | 6 | 143592386 | A | G | A/G | 0.561 | 0.201 | mmHg | 7.40E-15 | 321262 |
| BP | PP | rs7226020 | 17 | 6473828 | T | C | T/C | 0.563 | -0.232 | mmHg | 2.10E-17 | 321262 |
| BP | SBP | rs55940751 | 4 | 77365891 | C | T | C/T | 0.563 | 0.216 | mmHg | 3.60E-08 | 321262 |
| BP | SBP | rs303343 | 5 | 15312553 | C | T | C/T | 0.566 | -0.229 | mmHg | 3.50E-09 | 321262 |
| BP | SBP | rs9935770 | 16 | 21091291 | C | T | C/T | 0.568 | 0.223 | mmHg | 5.20E-09 | 321262 |
| BP | PP | rs9662255 | 1 | 9441949 | C | A | C/A | 0.574 | 0.177 | mmHg | 3.50E-10 | 321262 |
| BP | PP | rs1966203 | 10 | 21057545 | C | G | C/G | 0.576 | -0.143 | mmHg | 1.80E-08 | 321262 |
| BP | SBP | rs937213 | 15 | 40322124 | T | C | T/C | 0.577 | 0.256 | mmHg | 1.60E-10 | 321262 |
| BP | DBP | rs34331990 | 19 | 30321561 | T | G | T/G | 0.581 | -0.154 | mmHg | 2.80E-10 | 321262 |
| BP | SBP | rs4141663 | 3 | 124551967 | C | T | C/T | 0.582 | 0.258 | mmHg | 1.70E-11 | 321262 |
| BP | DBP | rs10103353 | 8 | 82849452 | C | T | C/T | 0.583 | 0.147 | mmHg | 4.30E-10 | 321262 |
| BP | PP | rs28594215 | 8 | 32395518 | G | A | G/A | 0.584 | -0.144 | mmHg | 2.10E-08 | 321262 |
| BP | PP | rs10958717 | 8 | 42351585 | G | C | G/C | 0.591 | -0.168 | mmHg | 1.00E-10 | 321262 |
| BP | DBP | rs6795735 | 3 | 64705365 | C | T | C/T | 0.591 | 0.159 | mmHg | 1.30E-11 | 321262 |
| BP | PP | rs9349379 | 6 | 12903957 | A | G | A/G | 0.594 | 0.195 | mmHg | 6.30E-13 | 321262 |
| BP | DBP | rs4686683 | 3 | 185307363 | T | G | T/G | 0.596 | -0.172 | mmHg | 3.80E-13 | 321262 |
| BP | DBP | rs4292285 | 4 | 145271954 | T | A | T/A | 0.598 | 0.154 | mmHg | 6.90E-11 | 321262 |
| BP | SBP | rs4292285 | 4 | 145271954 | T | A | T/A | 0.598 | 0.23 | mmHg | 3.00E-09 | 321262 |
| BP | DBP | rs1848797 | 10 | 64552934 | A | G | A/G | 0.601 | 0.191 | mmHg | 1.40E-15 | 321262 |
| BP | SBP | rs1848797 | 10 | 64552934 | A | G | A/G | 0.601 | 0.265 | mmHg | 1.40E-11 | 321262 |
| BP | PP | rs17471509 | 4 | 48301691 | A | G | A/G | 0.608 | 0.143 | mmHg | 3.50E-08 | 321262 |
| BP | PP | rs917275 | 7 | 28658522 | A | G | A/G | 0.609 | -0.182 | mmHg | 7.50E-12 | 321262 |
| BP | SBP | rs112875651 | 8 | 126506694 | G | A | G/A | 0.609 | 0.226 | mmHg | 2.30E-08 | 321262 |
| BP | DBP | rs11556924 | 7 | 129663496 | C | T | C/T | 0.611 | 0.181 | mmHg | 7.50E-13 | 321262 |
| BP | DBP | rs4923910 | 15 | 42086340 | G | C | G/C | 0.625 | -0.178 | mmHg | 3.50E-13 | 321262 |
| BP | PP | rs367700296 | 14 | 98597422 | G | A | G/GA | 0.635 | -0.274 | mmHg | 9.80E-09 | 321262 |
| BP | SBP | rs8904 | 14 | 35871217 | G | A | G/A | 0.636 | -0.259 | mmHg | 4.20E-11 | 321262 |
| BP | PP | rs8904 | 14 | 35871217 | G | A | G/A | 0.636 | -0.167 | mmHg | 1.50E-10 | 321262 |
| BP | DBP | rs8105753 | 19 | 31927547 | A | C | A/C | 0.638 | 0.137 | mmHg | 1.90E-08 | 321262 |
| BP | SBP | rs8105753 | 19 | 31927547 | A | C | A/C | 0.638 | 0.249 | mmHg | 3.60E-10 | 321262 |
| BP | PP | rs670463 | 6 | 53977495 | A | G | A/G | 0.644 | -0.158 | mmHg | 2.10E-09 | 321262 |
| BP | PP | rs7519279 | 1 | 169207361 | G | C | G/C | 0.644 | 0.21 | mmHg | 1.10E-13 | 321262 |
| BP | PP | rs11708647 | 3 | 142617353 | G | A | G/A | 0.646 | 0.178 | mmHg | 4.40E-11 | 321262 |
| BP | SBP | rs12504699 | 4 | 48934298 | G | A | G/A | 0.648 | 0.221 | mmHg | 2.10E-08 | 321262 |
| BP | PP | rs2540950 | 2 | 65279223 | C | T | C/T | 0.652 | 0.172 | mmHg | 6.30E-11 | 321262 |
| BP | SBP | rs7927515 | 11 | 76125330 | C | A | C/A | 0.654 | -0.235 | mmHg | 4.40E-09 | 321262 |
| BP | PP | rs7927515 | 11 | 76125330 | C | A | C/A | 0.654 | -0.149 | mmHg | 2.60E-08 | 321262 |
| BP | PP | rs17831815 | 3 | 66437086 | T | C | T/C | 0.654 | 0.176 | mmHg | 4.70E-11 | 321262 |
| BP | SBP | rs2585810 | 11 | 28483787 | G | A | G/A | 0.656 | -0.22 | mmHg | 2.70E-08 | 321262 |
| BP | PP | rs9337951 | 10 | 30317073 | G | A | G/A | 0.657 | -0.265 | mmHg | 1.00E-19 | 321262 |
| BP | SBP | rs2178452 | 3 | 160370160 | G | A | G/A | 0.658 | 0.259 | mmHg | 1.10E-10 | 321262 |
| BP | PP | rs12208834 | 6 | 157287299 | A | G | A/G | 0.668 | -0.15 | mmHg | 4.20E-08 | 321262 |
| BP | DBP | rs7734334 | 5 | 131815004 | C | A | C/A | 0.67 | -0.154 | mmHg | 8.70E-10 | 321262 |
| BP | PP | rs2012714 | 22 | 19977647 | C | T | C/T | 0.671 | -0.182 | mmHg | 2.80E-09 | 321262 |
| BP | DBP | rs1152958 | 12 | 70325669 | G | A | G/A | 0.674 | 0.138 | mmHg | 4.00E-08 | 321262 |
| BP | PP | rs956006 | 15 | 62808539 | C | T | C/T | 0.674 | 0.215 | mmHg | 2.90E-15 | 321262 |
| BP | PP | rs2244643 | 14 | 92359022 | A | C | A/C | 0.675 | -0.219 | mmHg | 4.00E-14 | 321262 |
| BP | DBP | rs3749237 | 3 | 49770032 | G | A | G/A | 0.675 | -0.165 | mmHg | 3.50E-11 | 321262 |
| BP | DBP | rs12787709 | 11 | 14639257 | G | A | G/A | 0.675 | -0.138 | mmHg | 3.40E-08 | 321262 |
| BP | DBP | rs4972805 | 2 | 177012570 | C | T | C/T | 0.677 | 0.143 | mmHg | 1.80E-08 | 321262 |
| BP | DBP | rs12906962 | 15 | 95312071 | T | C | T/C | 0.678 | -0.16 | mmHg | 1.30E-10 | 321262 |
| BP | DBP | rs67833703 | 17 | 3888437 | C | T | C/T | 0.679 | -0.147 | mmHg | 1.10E-08 | 321262 |
| BP | DBP | rs6429422 | 1 | 243472801 | T | G | T/G | 0.682 | -0.249 | mmHg | 1.50E-23 | 321262 |
| BP | SBP | rs6803322 | 3 | 84986088 | C | A | C/A | 0.682 | 0.228 | mmHg | 4.60E-08 | 321262 |
| BP | PP | rs11677932 | 2 | 238223955 | G | A | G/A | 0.683 | 0.151 | mmHg | 4.50E-08 | 321262 |
| BP | PP | rs3915499 | 16 | 15910743 | G | A | G/A | 0.683 | 0.152 | mmHg | 2.30E-08 | 321262 |
| BP | DBP | rs1053711 | 3 | 57743246 | G | A | G/A | 0.685 | 0.153 | mmHg | 2.20E-09 | 321262 |
| BP | SBP | rs12636552 | 3 | 70972466 | A | G | A/G | 0.686 | 0.241 | mmHg | 4.80E-09 | 321262 |
| BP | SBP | rs3731818 | 2 | 86368804 | G | A | G/A | 0.687 | 0.24 | mmHg | 4.10E-09 | 321262 |
| BP | DBP | rs11030119 | 11 | 27728102 | G | A | G/A | 0.693 | 0.18 | mmHg | 2.30E-12 | 321262 |
| BP | PP | rs12606620 | 18 | 42008097 | G | T | G/T | 0.694 | 0.229 | mmHg | 1.50E-16 | 321262 |
| BP | SBP | rs12606620 | 18 | 42008097 | G | T | G/T | 0.694 | 0.292 | mmHg | 2.50E-12 | 321262 |
| BP | SBP | rs6595838 | 5 | 127868199 | G | A | G/A | 0.696 | -0.267 | mmHg | 1.80E-10 | 321262 |
| BP | SBP | rs7041664 | 9 | 8010674 | C | A | C/A | 0.697 | -0.247 | mmHg | 7.10E-09 | 321262 |
| BP | PP | rs7041664 | 9 | 8010674 | C | A | C/A | 0.697 | -0.166 | mmHg | 5.60E-09 | 321262 |
| BP | PP | rs12248718 | 10 | 12242326 | A | G | A/G | 0.698 | -0.154 | mmHg | 4.60E-08 | 321262 |
| BP | PP | rs9729719 | 1 | 38298207 | G | A | G/A | 0.707 | -0.188 | mmHg | 2.00E-11 | 321262 |
| BP | DBP | rs8139817 | 22 | 18468369 | A | C | A/C | 0.708 | -0.143 | mmHg | 3.80E-08 | 321262 |
| BP | PP | rs2645466 | 17 | 57853214 | A | C | A/C | 0.709 | -0.152 | mmHg | 3.00E-08 | 321262 |
| BP | SBP | rs60199046 | 1 | 59663341 | A | G | A/G | 0.709 | 0.24 | mmHg | 2.60E-08 | 321262 |
| BP | PP | rs60199046 | 1 | 59663341 | A | G | A/G | 0.709 | 0.313 | mmHg | 1.30E-27 | 321262 |
| BP | SBP | rs34872471 | 10 | 114754071 | T | C | T/C | 0.71 | -0.25 | mmHg | 3.70E-09 | 321262 |
| BP | PP | rs34872471 | 10 | 114754071 | T | C | T/C | 0.71 | -0.204 | mmHg | 5.40E-13 | 321262 |
| BP | PP | rs649472 | 6 | 42673015 | T | C | T/C | 0.714 | 0.161 | mmHg | 1.20E-08 | 321262 |
| BP | PP | rs4980877 | 12 | 418916 | C | T | C/T | 0.716 | -0.162 | mmHg | 2.70E-08 | 321262 |
| BP | DBP | rs72812846 | 5 | 173377636 | T | A | T/A | 0.719 | 0.202 | mmHg | 1.00E-13 | 321262 |
| BP | DBP | rs12928482 | 16 | 81513871 | G | A | G/A | 0.721 | 0.147 | mmHg | 1.90E-08 | 321262 |
| BP | DBP | rs13403122 | 2 | 43078758 | C | T | C/T | 0.722 | 0.195 | mmHg | 1.90E-13 | 321262 |
| BP | SBP | rs13403122 | 2 | 43078758 | C | T | C/T | 0.722 | 0.25 | mmHg | 8.90E-09 | 321262 |
| BP | PP | rs3790227 | 20 | 19469002 | C | A | C/A | 0.723 | -0.22 | mmHg | 3.00E-15 | 321262 |
| BP | DBP | rs2246438 | 10 | 45273079 | G | A | G/A | 0.726 | 0.16 | mmHg | 1.40E-09 | 321262 |
| BP | DBP | rs9845655 | 3 | 56701328 | T | C | T/C | 0.726 | 0.161 | mmHg | 5.00E-10 | 321262 |
| BP | DBP | rs17046596 | 1 | 217722449 | A | C | A/C | 0.743 | -0.151 | mmHg | 1.50E-08 | 321262 |
| BP | PP | rs2215590 | 14 | 73297741 | C | T | C/T | 0.745 | -0.161 | mmHg | 1.80E-08 | 321262 |
| BP | SBP | rs7008914 | 8 | 25880400 | T | C | T/C | 0.746 | 0.238 | mmHg | 4.50E-08 | 321262 |
| BP | PP | rs7500448 | 16 | 83045790 | A | G | A/G | 0.746 | 0.301 | mmHg | 3.30E-24 | 321262 |
| BP | SBP | rs13050325 | 21 | 16343812 | A | G | A/G | 0.747 | -0.258 | mmHg | 5.50E-09 | 321262 |
| BP | PP | rs34489224 | 7 | 100525559 | C | G | C/G | 0.756 | 0.185 | mmHg | 3.80E-10 | 321262 |
| BP | DBP | rs36061333 | 6 | 116311763 | C | G | C/G | 0.758 | 0.16 | mmHg | 2.10E-08 | 321262 |
| BP | DBP | rs2280861 | 8 | 23404785 | A | G | A/G | 0.759 | -0.173 | mmHg | 6.70E-11 | 321262 |
| BP | DBP | rs72799341 | 16 | 30936743 | G | A | G/A | 0.759 | -0.161 | mmHg | 3.70E-09 | 321262 |
| BP | PP | rs11008355 | 10 | 31412561 | G | C | G/C | 0.76 | 0.168 | mmHg | 9.90E-09 | 321262 |
| BP | PP | rs1938598 | 11 | 58413910 | T | C | T/C | 0.76 | 0.21 | mmHg | 1.20E-12 | 321262 |
| BP | SBP | rs1938598 | 11 | 58413910 | T | C | T/C | 0.76 | 0.33 | mmHg | 1.10E-13 | 321262 |
| BP | DBP | rs11168244 | 12 | 48202941 | C | T | C/T | 0.761 | 0.165 | mmHg | 4.70E-08 | 321262 |
| BP | SBP | rs11168244 | 12 | 48202941 | C | T | C/T | 0.761 | 0.318 | mmHg | 2.10E-10 | 321262 |
| BP | DBP | rs35189230 | 6 | 117816351 | G | A | G/GCA | 0.763 | 0.239 | mmHg | 1.00E-08 | 321262 |
| BP | DBP | rs58117425 | 2 | 145681570 | G | A | G/A | 0.764 | -0.192 | mmHg | 8.30E-12 | 321262 |
| BP | SBP | rs9314907 | 13 | 115015163 | C | T | C/T | 0.765 | -0.292 | mmHg | 1.30E-10 | 321262 |
| BP | PP | rs13420463 | 2 | 37517566 | A | G | A/G | 0.77 | 0.159 | mmHg | 4.90E-08 | 321262 |
| BP | SBP | rs13420463 | 2 | 37517566 | A | G | A/G | 0.77 | 0.282 | mmHg | 1.40E-10 | 321262 |
| BP | PP | rs11638064 | 15 | 67460009 | G | A | G/A | 0.774 | -0.168 | mmHg | 1.70E-08 | 321262 |
| BP | DBP | rs2034618 | 15 | 83799632 | C | T | C/T | 0.779 | 0.188 | mmHg | 2.00E-11 | 321262 |
| BP | PP | rs6747874 | 2 | 101578489 | G | A | G/A | 0.782 | -0.171 | mmHg | 1.20E-08 | 321262 |
| BP | PP | rs2971669 | 7 | 44231778 | C | T | C/T | 0.782 | -0.169 | mmHg | 3.40E-08 | 321262 |
| BP | PP | rs2424908 | 20 | 31360383 | C | T | C/T | 0.783 | -0.189 | mmHg | 4.50E-10 | 321262 |
| BP | PP | rs73605614 | 10 | 20529470 | A | C | A/C | 0.783 | -0.177 | mmHg | 1.20E-08 | 321262 |
| BP | DBP | rs1544935 | 6 | 39124448 | T | G | T/G | 0.785 | 0.176 | mmHg | 8.90E-10 | 321262 |
| BP | DBP | rs17617337 | 10 | 121426884 | C | T | C/T | 0.786 | 0.172 | mmHg | 3.10E-09 | 321262 |
| BP | PP | rs57400569 | 4 | 89752276 | G | A | G/A | 0.786 | 0.204 | mmHg | 1.30E-11 | 321262 |
| BP | PP | rs7914287 | 10 | 69350563 | T | C | T/C | 0.788 | -0.187 | mmHg | 5.70E-10 | 321262 |
| BP | DBP | rs4808569 | 19 | 17218970 | C | A | C/A | 0.797 | 0.162 | mmHg | 4.50E-08 | 321262 |
| BP | DBP | rs7980687 | 12 | 123822711 | G | A | G/A | 0.797 | 0.196 | mmHg | 1.70E-11 | 321262 |
| BP | PP | rs1261744 | 11 | 117218460 | T | C | T/C | 0.8 | -0.264 | mmHg | 7.30E-17 | 321262 |
| BP | PP | rs72958213 | 2 | 217646523 | C | T | C/T | 0.803 | 0.177 | mmHg | 4.50E-08 | 321262 |
| BP | DBP | rs34594435 | 7 | 72977249 | C | T | C/T | 0.804 | 0.183 | mmHg | 1.30E-09 | 321262 |
| BP | PP | rs200688233 | 19 | 10372360 | G | C | G/C | 0.807 | -0.314 | mmHg | 3.90E-08 | 321262 |
| BP | PP | rs11222386 | 11 | 130779068 | G | C | G/C | 0.808 | -0.178 | mmHg | 4.40E-08 | 321262 |
| BP | PP | rs169287 | 6 | 27854760 | C | A | C/A | 0.809 | -0.19 | mmHg | 3.20E-08 | 321262 |
| BP | DBP | rs169287 | 6 | 27854760 | C | A | C/A | 0.809 | 0.215 | mmHg | 5.10E-12 | 321262 |
| BP | PP | rs3820068 | 1 | 15798197 | A | G | A/G | 0.81 | 0.199 | mmHg | 1.10E-09 | 321262 |
| BP | SBP | rs35410524 | 6 | 96885405 | C | T | C/T | 0.815 | -0.305 | mmHg | 4.70E-10 | 321262 |
| BP | DBP | rs507666 | 9 | 136149399 | G | A | G/A | 0.815 | 0.169 | mmHg | 1.70E-08 | 321262 |
| BP | DBP | rs6129880 | 20 | 40251829 | T | G | T/G | 0.815 | 0.174 | mmHg | 2.10E-09 | 321262 |
| BP | SBP | rs6129880 | 20 | 40251829 | T | G | T/G | 0.815 | 0.264 | mmHg | 2.10E-08 | 321262 |
| BP | PP | rs137993948 | 1 | 23369277 | T | G | T/G | 0.822 | -0.196 | mmHg | 1.00E-08 | 321262 |
| BP | PP | rs2193635 | 18 | 43096236 | C | T | C/T | 0.823 | -0.324 | mmHg | 9.30E-24 | 321262 |
| BP | SBP | rs2193635 | 18 | 43096236 | C | T | C/T | 0.823 | -0.274 | mmHg | 1.60E-08 | 321262 |
| BP | PP | rs7838781 | 8 | 77588716 | A | G | A/G | 0.826 | 0.183 | mmHg | 1.90E-08 | 321262 |
| BP | PP | rs12037669 | 1 | 201721930 | T | G | T/G | 0.826 | 0.22 | mmHg | 1.40E-10 | 321262 |
| BP | SBP | rs11993898 | 8 | 51936632 | T | C | T/C | 0.828 | -0.326 | mmHg | 3.50E-11 | 321262 |
| BP | PP | rs6782694 | 3 | 141627860 | C | A | C/A | 0.828 | -0.19 | mmHg | 2.10E-08 | 321262 |
| BP | DBP | rs11021221 | 11 | 95308854 | T | A | T/A | 0.832 | 0.213 | mmHg | 2.70E-11 | 321262 |
| BP | DBP | rs6428947 | 1 | 236326005 | C | G | C/G | 0.833 | 0.205 | mmHg | 3.20E-11 | 321262 |
| BP | DBP | rs9864898 | 3 | 138111751 | C | T | C/T | 0.84 | -0.19 | mmHg | 4.60E-09 | 321262 |
| BP | PP | rs62361303 | 5 | 108102727 | C | T | C/T | 0.843 | 0.235 | mmHg | 1.90E-11 | 321262 |
| BP | DBP | rs6060114 | 20 | 30169673 | T | C | T/C | 0.845 | 0.237 | mmHg | 6.50E-13 | 321262 |
| BP | SBP | rs13024657 | 2 | 175472839 | C | T | C/T | 0.847 | -0.296 | mmHg | 3.60E-08 | 321262 |
| BP | PP | rs13024657 | 2 | 175472839 | C | T | C/T | 0.847 | -0.216 | mmHg | 1.90E-09 | 321262 |
| BP | DBP | rs11486794 | 7 | 2491918 | C | T | C/T | 0.848 | 0.219 | mmHg | 7.70E-10 | 321262 |
| BP | PP | rs62011052 | 15 | 79156983 | T | C | T/C | 0.849 | -0.28 | mmHg | 3.10E-15 | 321262 |
| BP | PP | rs6793656 | 3 | 13823342 | A | G | A/G | 0.849 | -0.224 | mmHg | 5.70E-10 | 321262 |
| BP | PP | rs139703184 | 9 | 127937746 | T | A | T/A | 0.85 | -0.271 | mmHg | 1.10E-13 | 321262 |
| BP | PP | rs36010659 | 18 | 48283949 | T | C | T/C | 0.857 | 0.254 | mmHg | 4.00E-12 | 321262 |
| BP | SBP | rs1878406 | 4 | 148393664 | C | T | C/T | 0.859 | -0.321 | mmHg | 2.00E-09 | 321262 |
| BP | PP | rs1878406 | 4 | 148393664 | C | T | C/T | 0.859 | -0.292 | mmHg | 4.40E-16 | 321262 |
| BP | PP | rs57448815 | 21 | 30123533 | A | G | A/G | 0.86 | -0.227 | mmHg | 6.00E-09 | 321262 |
| BP | DBP | rs2236973 | 3 | 50474284 | T | C | T/C | 0.86 | -0.182 | mmHg | 4.90E-08 | 321262 |
| BP | DBP | rs17286052 | 5 | 87430302 | A | G | A/G | 0.86 | 0.188 | mmHg | 2.60E-08 | 321262 |
| BP | SBP | rs17286052 | 5 | 87430302 | A | G | A/G | 0.86 | 0.36 | mmHg | 6.50E-11 | 321262 |
| BP | PP | rs10199082 | 2 | 56040099 | T | C | T/C | 0.864 | 0.26 | mmHg | 1.70E-11 | 321262 |
| BP | DBP | rs10427021 | 19 | 7259346 | T | G | T/G | 0.864 | 0.343 | mmHg | 4.50E-22 | 321262 |
| BP | SBP | rs10427021 | 19 | 7259346 | T | G | T/G | 0.864 | 0.512 | mmHg | 9.50E-19 | 321262 |
| BP | SBP | rs34887403 | 22 | 29151150 | G | A | G/A | 0.865 | -0.304 | mmHg | 3.10E-08 | 321262 |
| BP | DBP | rs4709746 | 6 | 164133001 | C | T | C/T | 0.867 | 0.19 | mmHg | 4.10E-08 | 321262 |
| BP | SBP | rs74237369 | 11 | 55355182 | G | T | G/T | 0.868 | 0.315 | mmHg | 4.50E-08 | 321262 |
| BP | SBP | rs9565436 | 13 | 36213631 | A | C | A/C | 0.871 | -0.303 | mmHg | 2.60E-08 | 321262 |
| BP | SBP | rs61448762 | 11 | 48923756 | G | A | G/A | 0.872 | 0.358 | mmHg | 3.50E-09 | 321262 |
| BP | PP | rs114407963 | 2 | 204154677 | A | T | A/T | 0.875 | -0.234 | mmHg | 2.50E-09 | 321262 |
| BP | DBP | rs386459788^*^ | 7 | 80387316 | T | C | T/C | 0.876 | 0.216 | mmHg | 1.10E-09 | 321262 |
| BP | PP | rs10818775 | 9 | 125755571 | C | T | C/T | 0.881 | 0.263 | mmHg | 1.90E-12 | 321262 |
| BP | SBP | rs10818775 | 9 | 125755571 | C | T | C/T | 0.881 | 0.332 | mmHg | 3.70E-09 | 321262 |
| BP | SBP | rs17423264 | 7 | 108090255 | C | T | C/T | 0.881 | 0.366 | mmHg | 4.90E-08 | 321262 |
| BP | PP | rs11690961 | 2 | 46363336 | A | C | A/C | 0.883 | 0.313 | mmHg | 1.20E-14 | 321262 |
| BP | SBP | rs12670854 | 7 | 1731866 | A | G | A/G | 0.89 | 0.36 | mmHg | 1.20E-08 | 321262 |
| BP | PP | rs560276033 | 7 | 91268940 | G | G | GTA/G | 0.892 | -0.429 | mmHg | 1.60E-09 | 321262 |
| BP | DBP | rs72930293 | 11 | 69073420 | C | T | C/T | 0.892 | 0.234 | mmHg | 1.70E-09 | 321262 |
| BP | PP | rs7977389 | 12 | 49981722 | T | C | T/C | 0.894 | 0.274 | mmHg | 1.10E-11 | 321262 |
| BP | SBP | rs7977389 | 12 | 49981722 | T | C | T/C | 0.894 | 0.381 | mmHg | 3.30E-10 | 321262 |
| BP | PP | rs13192976 | 6 | 152312415 | A | T | A/T | 0.896 | -0.43 | mmHg | 8.20E-28 | 321262 |
| BP | PP | rs12538229 | 7 | 40460129 | C | T | C/T | 0.896 | 0.289 | mmHg | 2.40E-12 | 321262 |
| BP | DBP | rs113161639 | 3 | 154615819 | G | T | G/T | 0.899 | 0.288 | mmHg | 3.20E-13 | 321262 |
| BP | SBP | rs113161639 | 3 | 154615819 | G | T | G/T | 0.899 | 0.451 | mmHg | 3.90E-12 | 321262 |
| BP | PP | rs2498323 | 4 | 3451109 | G | A | G/A | 0.9 | -0.29 | mmHg | 1.10E-09 | 321262 |
| BP | PP | rs12206253 | 6 | 122192592 | C | T | C/T | 0.902 | 0.287 | mmHg | 1.10E-12 | 321262 |
| BP | PP | rs2404715 | 1 | 57008778 | C | T | C/T | 0.908 | 0.338 | mmHg | 2.70E-14 | 321262 |
| BP | SBP | rs2404715 | 1 | 57008778 | C | T | C/T | 0.908 | 0.4 | mmHg | 1.70E-09 | 321262 |
| BP | PP | rs74482535 | 11 | 44030783 | C | T | C/T | 0.91 | 0.271 | mmHg | 1.00E-09 | 321262 |
| BP | PP | rs12485003 | 22 | 40635276 | G | A | G/A | 0.912 | -0.327 | mmHg | 4.60E-13 | 321262 |
| BP | PP | rs147696085 | 1 | 51021867 | G | A | G/A | 0.912 | 0.29 | mmHg | 2.20E-11 | 321262 |
| BP | PP | rs9708177 | 15 | 90649072 | C | T | C/T | 0.917 | -0.274 | mmHg | 4.30E-08 | 321262 |
| BP | PP | rs76785029 | 12 | 94882905 | C | T | C/T | 0.918 | 0.379 | mmHg | 5.40E-14 | 321262 |
| BP | SBP | rs80073370 | 8 | 19833156 | A | T | A/T | 0.918 | 0.404 | mmHg | 8.40E-09 | 321262 |
| BP | SBP | rs76735299 | 8 | 142396481 | G | A | G/A | 0.921 | -0.429 | mmHg | 3.60E-08 | 321262 |
| BP | DBP | rs1630266 | 6 | 118612943 | G | A | G/A | 0.921 | -0.252 | mmHg | 2.80E-09 | 321262 |
| BP | PP | rs734780 | 15 | 89564958 | T | C | T/C | 0.925 | 0.245 | mmHg | 1.60E-08 | 321262 |
| BP | SBP | rs9844972 | 3 | 150097635 | G | C | G/C | 0.93 | -0.441 | mmHg | 8.90E-09 | 321262 |
| BP | PP | rs9844972 | 3 | 150097635 | G | C | G/C | 0.93 | -0.296 | mmHg | 7.40E-09 | 321262 |
| BP | DBP | rs36226649 | 14 | 24835500 | T | C | T/C | 0.933 | -0.297 | mmHg | 2.60E-09 | 321262 |
| BP | PP | rs7312132 | 12 | 110352509 | G | C | G/C | 0.934 | 0.283 | mmHg | 6.80E-09 | 321262 |
| BP | DBP | rs903432 | 6 | 166175471 | A | G | A/G | 0.936 | 0.313 | mmHg | 5.10E-12 | 321262 |
| BP | DBP | rs3923097 | 2 | 124020790 | T | A | T/A | 0.944 | 0.273 | mmHg | 1.40E-08 | 321262 |
| BP | PP | rs61823001 | 1 | 176664440 | A | G | A/G | 0.948 | 0.312 | mmHg | 4.40E-08 | 321262 |
| BP | DBP | rs111630016 | 7 | 158048396 | C | T | C/T | 0.948 | 0.324 | mmHg | 3.20E-08 | 321262 |
| BP | DBP | rs76398786 | 3 | 48731450 | C | T | C/T | 0.949 | -0.382 | mmHg | 1.50E-08 | 321262 |
| BP | PP | rs74621754 | 3 | 8496371 | G | A | G/A | 0.952 | 0.36 | mmHg | 2.70E-08 | 321262 |
| BP | DBP | rs17210898 | 12 | 51056511 | G | A | G/A | 0.959 | 0.351 | mmHg | 1.10E-08 | 321262 |
| BP | SBP | rs17210898 | 12 | 51056511 | G | A | G/A | 0.959 | 0.556 | mmHg | 3.50E-08 | 321262 |
| BP | DBP | rs111304266 | 5 | 56589542 | C | G | C/G | 0.96 | -0.37 | mmHg | 3.30E-08 | 321262 |
| BP | DBP | rs112204826 | 21 | 44721027 | C | T | C/T | 0.962 | -0.395 | mmHg | 2.70E-08 | 321262 |
| BP | SBP | rs28663144 | 9 | 113198891 | A | C | A/C | 0.965 | -0.694 | mmHg | 3.50E-11 | 321262 |
| BP | DBP | rs17082391 | 5 | 91900785 | C | G | C/G | 0.969 | 0.375 | mmHg | 1.30E-08 | 321262 |
| BP | PP | rs62270945 | 3 | 128201889 | C | T | C/T | 0.972 | -0.534 | mmHg | 1.70E-10 | 321262 |
| BP | PP | rs61735998 | 18 | 34289285 | G | T | G/T | 0.975 | -0.519 | mmHg | 2.40E-08 | 321262 |
| BP | DBP | rs117638970 | 15 | 69675605 | C | T | C/T | 0.975 | 0.484 | mmHg | 2.20E-08 | 321262 |
| BP | PP | rs117204111 | 11 | 118199425 | G | A | G/A | 0.976 | 0.491 | mmHg | 3.00E-08 | 321262 |
| BP | DBP | rs11102916 | 1 | 115836746 | C | A | C/A | 0.979 | -0.575 | mmHg | 9.50E-12 | 321262 |
| BP | PP | rs117870289 | 21 | 39983448 | C | T | C/T | 0.985 | -0.732 | mmHg | 2.40E-09 | 321262 |
| BP | DBP | rs78378222 | 17 | 7571752 | T | G | T/G | 0.988 | -0.638 | mmHg | 1.00E-08 | 321262 |
| BP | PP | rs78378222 | 17 | 7571752 | T | G | T/G | 0.988 | 0.875 | mmHg | 4.30E-13 | 321262 |
| BP | PP | rs143750586 | 12 | 4358078 | A | G | A/G | 0.992 | 1.038 | mmHg | 1.40E-08 | 321262 |
| BP | DBP | rs139491786 | 16 | 2086421 | C | T | C/T | 0.993 | 1.155 | mmHg | 1.20E-09 | 321262 |
| BP | SBP | rs148871069 | 5 | 159404471 | A | G | A/G | 0.994 | 2.37 | mmHg | 3.90E-11 | 321262 |
| BP | PP | rs4792830^’^ | 17 | 44199290 | T | C | T/C | NA | -0.242 | mmHg | 2.90E-08 | 321262 |
| BP | PP | rs4631439 | 8 | 141059650 | C | T | C/T | NA | 0.203 | mmHg | 2.20E-10 | 321262 |
| BP | DBP | rs9508495^’^ | 13 | 30146201 | C | T | C/T | NA | 0.24 | mmHg | 1.10E-11 | 321262 |
| BP | DBP | rs55688777^’^ | 3 | 133886705 | T | C | T/C | NA | 0.256 | mmHg | 7.00E-14 | 321262 |
| BP | SBP | rs63418562 | 13 | 30146201 | C | T | C/T | NA | 0.312 | mmHg | 8.90E-09 | 321262 |
| BP | SBP | rs75305034 | 3 | 133886705 | T | C | T/C | NA | 0.331 | mmHg | 3.40E-10 | 321262 |

* rs4792830- SNP name in Hoffmann et.al: rs115231027

rs9508495- SNP name in Hoffmann et. al: rs63418562

rs55688777- SNP name in Hoffmann et. al: rs75305034

## rs386459788- SNP name in Hoffmann et. al rs76627715
