# Supplementary material for: Hypertension genetic risk score is associated with burden of coronary heart disease among patients referred for coronary angiography
Source: PLoS One. 2018 Dec 19;13(12):e0208645. doi: 10.1371/journal.pone.0208645 (PMC6300273; doi:10.1371/journal.pone.0208645)
Supplement: S4 Table — (DOCX) [file pone.0208645.s005.docx]

**S4 Table. Results of ANOVA and generalized linear model (GLM) analysis for difference in hypertension genetic risk score according to number of affected coronary vessels**

| ANOVA |  |  |  |  |  |  |
| --- | --- | --- | --- | --- | --- | --- |
|  |  | Df | Sum Sq | Mean Sq | F value | P-value |
|  | Vessel disease | 3 | 3426 | 1142.1 | 6.675 | 0.000171 |
|  | Residuals | 4805 | 822131 | 171.1 |  |  |

| GLM |  |  |  |  |  |
| --- | --- | --- | --- | --- | --- |
|  |  |  |  |  |  |
| Deviance Residuals: |  |  |  |  |  |
|  | Min | 1Q | Median | 3Q | Max |
|  | -44.921 | -9.034 | 0.009 | 9.023 | 55.106 |
|  |  |  |  |  |  |
| Coefficients: |  |  |  |  |  |
|  | Estimate | Std. Error | t value | p value |  |
| (Intercept) | 397.7994 | 1.928472222 | 1432.580 | <2.00E-16 |  |
| 1-coronary vessel disease | 0.3627 | 0.4594 | 0.547916667 | 0.42987 |  |
| 2-coronary vessel disease | 1.5192 | 0.5766 | 0.524305556 | 0.00845 |  |
| 3-coronary vessel disease | 2.3601 | 0.5858 | 4.029 | 5.7e-05 |  |
|  |  |  |  |  |  |
|  |  |  |  |  |  |
| Null deviance: | 825557 on 4808 degrees of freedom |  |  |  |  |
| Residual deviance: | 822131 on 4805 degrees of freedom |  |  |  |  |
| AIC: | 38382 |  |  |  |  |
|  |  |  |  |  |  |
| Number of Fisher Scoring iterations: | 2 |  |  |  |  |
