# Supplementary material for: Hypertension genetic risk score is associated with burden of coronary heart disease among patients referred for coronary angiography
Source: PLoS One. 2018 Dec 19;13(12):e0208645. doi: 10.1371/journal.pone.0208645 (PMC6300273; doi:10.1371/journal.pone.0208645)
Supplement: S5 Table — (DOCX) [file pone.0208645.s006.docx]

**S5 Table. Demographics Inter99 (N=4,912).**

| Variable |  | Total |
| --- | --- | --- |
| Age | median [iqr] | 45.0 [39.9, 50.1] |
| Gender | Males | 2273 (46.3%) |
|  | Females | 2639 (53.7%) |
| uGRS | median [iqr] | 359.7 [350.9, 369.1] |
| wGRS | median [iqr] | 380.5 [372.3, 388.3] |
| Smoking |  | 3163 (64.4%) |
| BMI^a^ | Normal range | 2277 (46.4%) |
|  | Underweight | 58 (1.2%) |
|  | Overweight | 1863 (37.9%) |
|  | Obese | 714 (14.5%) |
| Hypertension |  | 1170 (23.8%) |
| Cholesterol^b^ | Normal range | 1635 (33.3%) |
|  | Mild hypercholesterolaemia | 2434 (49.6%) |
|  | Moderate hypercholesterolaemia | 759 (15.5%) |
|  | Severe hypercholesterolaemia | 84 (1.7%) |
| Diabetes |  | 309 (6.3%) |
| Coronary heart disease |  | 275 (5.6%) |
| Acute myocardial infarction |  | 91 (1.9%) |
| Stroke |  | 159 (3.2%) |

^a^BMI: Body mass index

Underweight (<18.5 kg/m^2^)

Normal weight (18.5-25 kg/m^2^)

Overweight (25.1-30 kg/m^2^)

Obese (>30 kg/m^2^)

^b^ Cholesterol: Mild hypercholesterolemia: 5.0-6.4 mmol/L

Moderate hypercholesterolemia: 6.5 mmol/L-7.9 mmol/L

Severe hypercholesterolemia: >=8.0 mmol/L
