# Supplementary material for: Hypertension genetic risk score is associated with burden of coronary heart disease among patients referred for coronary angiography
Source: PLoS One. 2018 Dec 19;13(12):e0208645. doi: 10.1371/journal.pone.0208645 (PMC6300273; doi:10.1371/journal.pone.0208645)
Supplement: S6 Table — (DOCX) [file pone.0208645.s007.docx]

**S6 Table. Evaluation of pleiotropy between hypertension GRS and BMI, hyperlipidemia, smoking and diabetes (Inter99) (N=4,192).**

| Hypertension GRS pleiotropy with BMI |  |  |  |  |  |  |  |  |  |  |  |
| --- | --- | --- | --- | --- | --- | --- | --- | --- | --- | --- | --- |
|  | Intercept | 95% CI | OR (2.Q.)^a^ | 95% CI | p-value | OR (3.Q.)^b^ | 95% CI | p-value | OR (4.Q.)^c^ | 95% CI | p-value |
| Underweight | 0.03 | 0.02-0.05 | 1.11 | 0.55-2.22 | 0.77 | 0.94 | 0.45-1.94 | 0.86 | 0.65 | 0.29-1.46 | 0.30 |
| Overweight | 0.90 | 0.80-1.02 | 0.84 | 0.71-1.00 | 0.05 | 0.89 | 0.75-1.06 | 0.18 | 0.90 | 0.76-1.07 | 0.24 |
| Obese | 0.32 | 0.27-0.38 | 0.89 | 0.70-1.13 | 0.35 | 0.89 | 0.70-1.13 | 0.35 | 1.09 | 0.87-1.38 | 0.45 |
|  |  |  |  |  |  |  |  |  |  |  |  |
| Hypertension GRS pleiotropy with hyperlipidemia |  |  |  |  |  |  |  |  |  |  |  |
|  | Intercept | 95% CI | OR (2.Q.) | 95% CI | p-value | OR (3.Q.) | 95% CI | p-value | OR (4.Q.) | 95% CI | p-value |
| Mild hyperlipidemia^d^ | 1.42 | 1.25-1.60 | 0.97 | 0.81-1.15 | 0.72 | 1.18 | 0.99-1.42 | 0.06 | 1.06 | 0.89-1.27 | 0.49 |
| Moderate hyperlipidemia^e^ | 0.45 | 0.38-0.53 | 0.94 | 0.74-1.20 | 0.62 | 1.12 | 0.87-1.42 | 0.38 | 1.08 | 0.85-1.38 | 0.53 |
| Severe hyperlipidemia^f^ | 0.05 | 0.03-0.08 | 0.98 | 0.53-1.82 | 0.94 | 1.21 | 0.66-2.34 | 0.53 | 0.95 | 0.50-1.80 | 0.88 |
|  |  |  |  |  |  |  |  |  |  |  |  |
| Hypertension GRS pleiotropy with smoking |  |  |  |  |  |  |  |  |  |  |  |
|  |  |  | OR (2.Q.) | 95% CI | p-value | OR (3.Q.) | 95% CI | p-value | OR (4.Q.) | 95% CI | p-value |
|  |  |  | 1.10 | 0.93-1.30 | 0.26 | 0.94 | 0.80-1.10 | 0.44 | 1.01 | 0.86-1.19 | 0.90 |
| Hypertension GRS pleiotropy with diabetes |  |  |  |  |  |  |  |  |  |  |  |
|  |  |  | OR (2.Q.) | 95% CI | p-value | OR (3.Q.) | 95% CI | p-value | OR (4.Q.) | 95% CI | p-value |
|  |  |  | 1.02 | 0.73-1.42 | 0.92 | 0.96 | 0.68-1.34 | 0.79 | 1.29 | 0.94-1.78 | 0.11 |

^a,b,c^Quantiles based on the number of hypertension increasing risk alleles: 1.Q. [314, 351]; 2.Q. (351, 360]; 3.Q. (360, 369]; 4.Q. (369, 406]

^d^Mild hypercholesterolemia: 5.0-6.4 mmol/L

^e^Moderate hypercholesterolemia: 6.5 mmol/L-7.9 mmol/L

^f^Severe hypercholesterolemia: >=8.0 mmol/L
