# Supplementary figures and images for: Hypertension genetic risk score is associated with burden of coronary heart disease among patients referred for coronary angiography
Source: PLoS One. 2018 Dec 19;13(12):e0208645. doi: 10.1371/journal.pone.0208645 (PMC6300273; doi:10.1371/journal.pone.0208645)

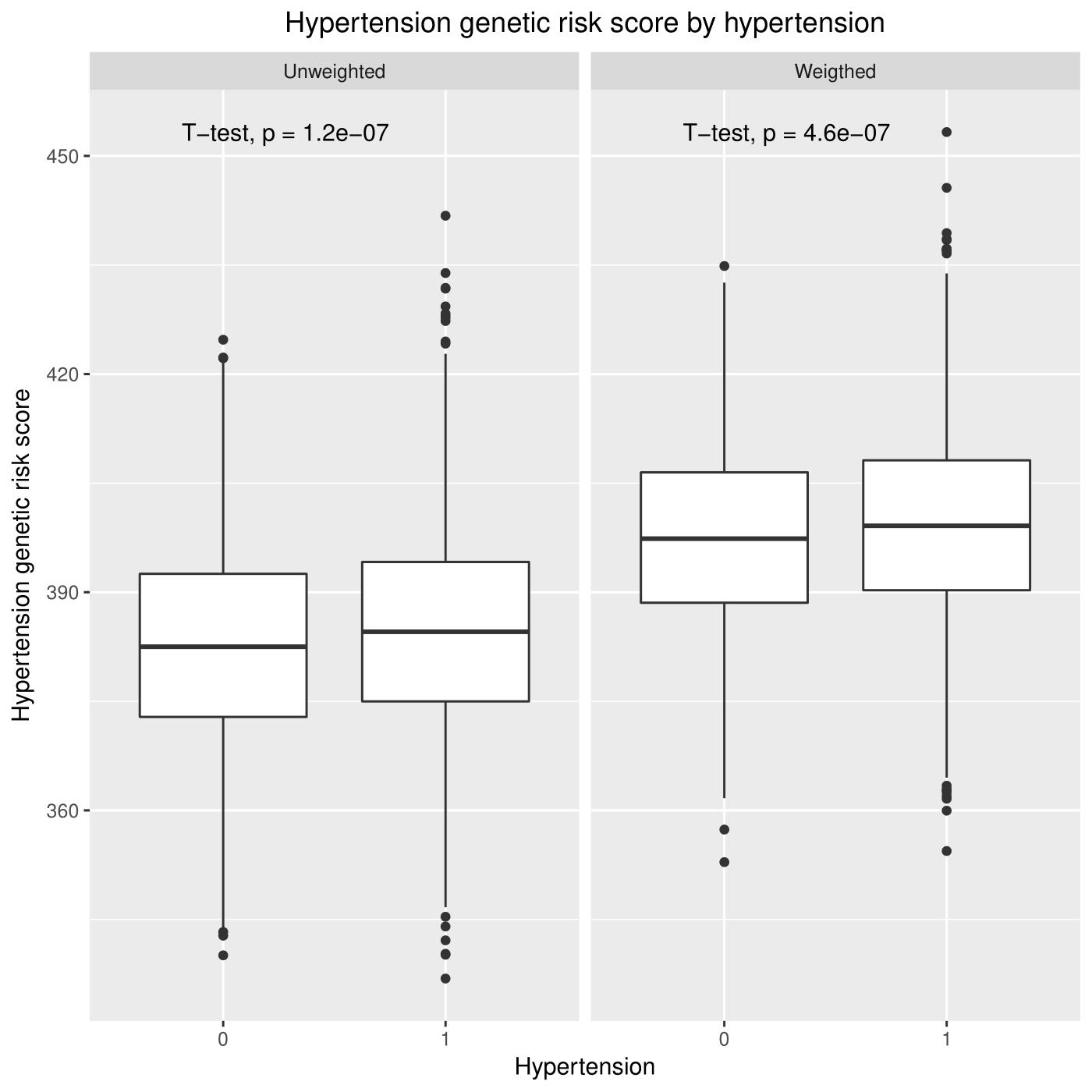

Supplement: S1 Fig — (TIFF) [file pone.0208645.s008.tiff]

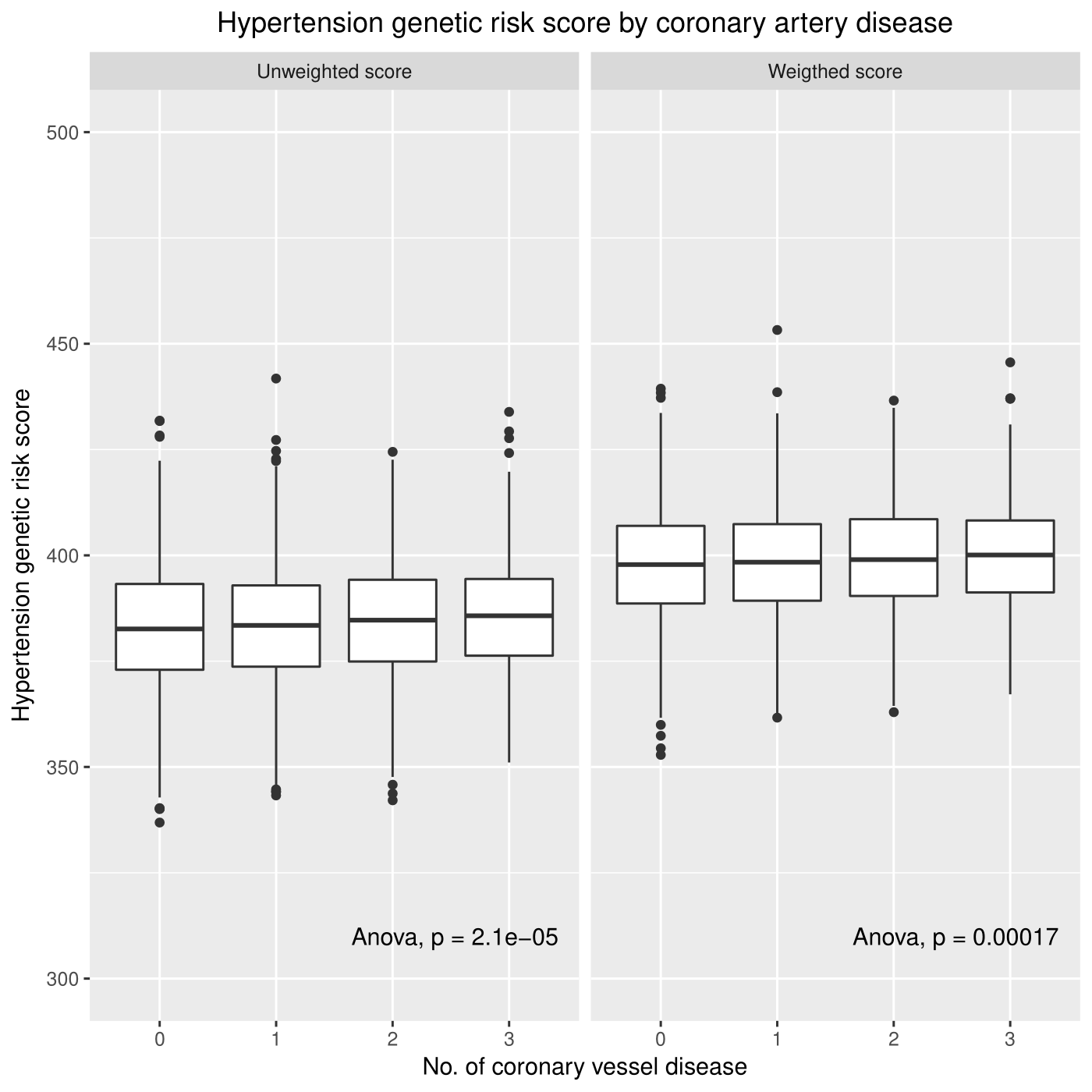

Supplement: S2 Fig — (TIFF) [file pone.0208645.s009.tiff]

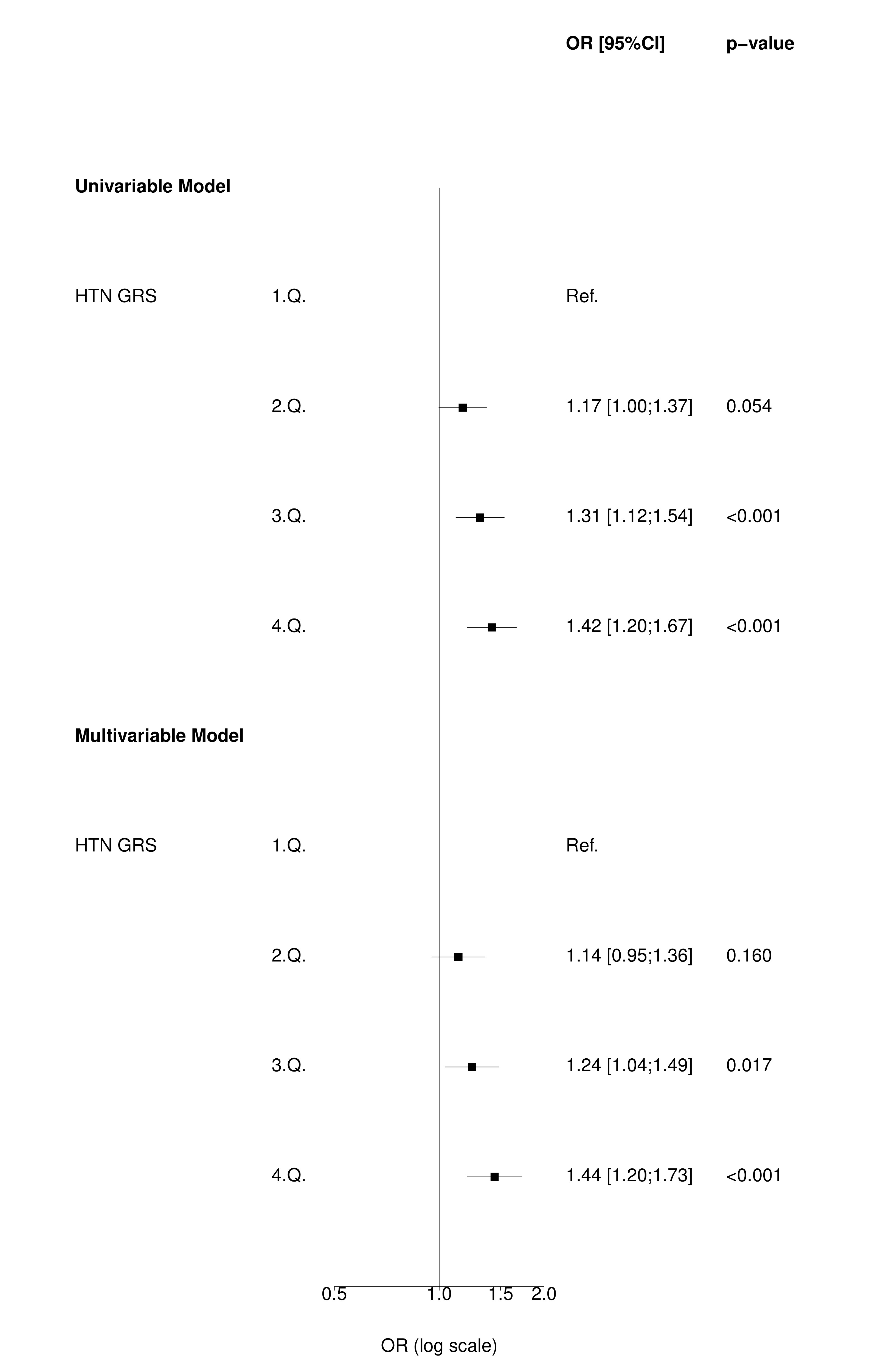

Supplement: S3 Fig — (TIFF) [file pone.0208645.s010.tiff]

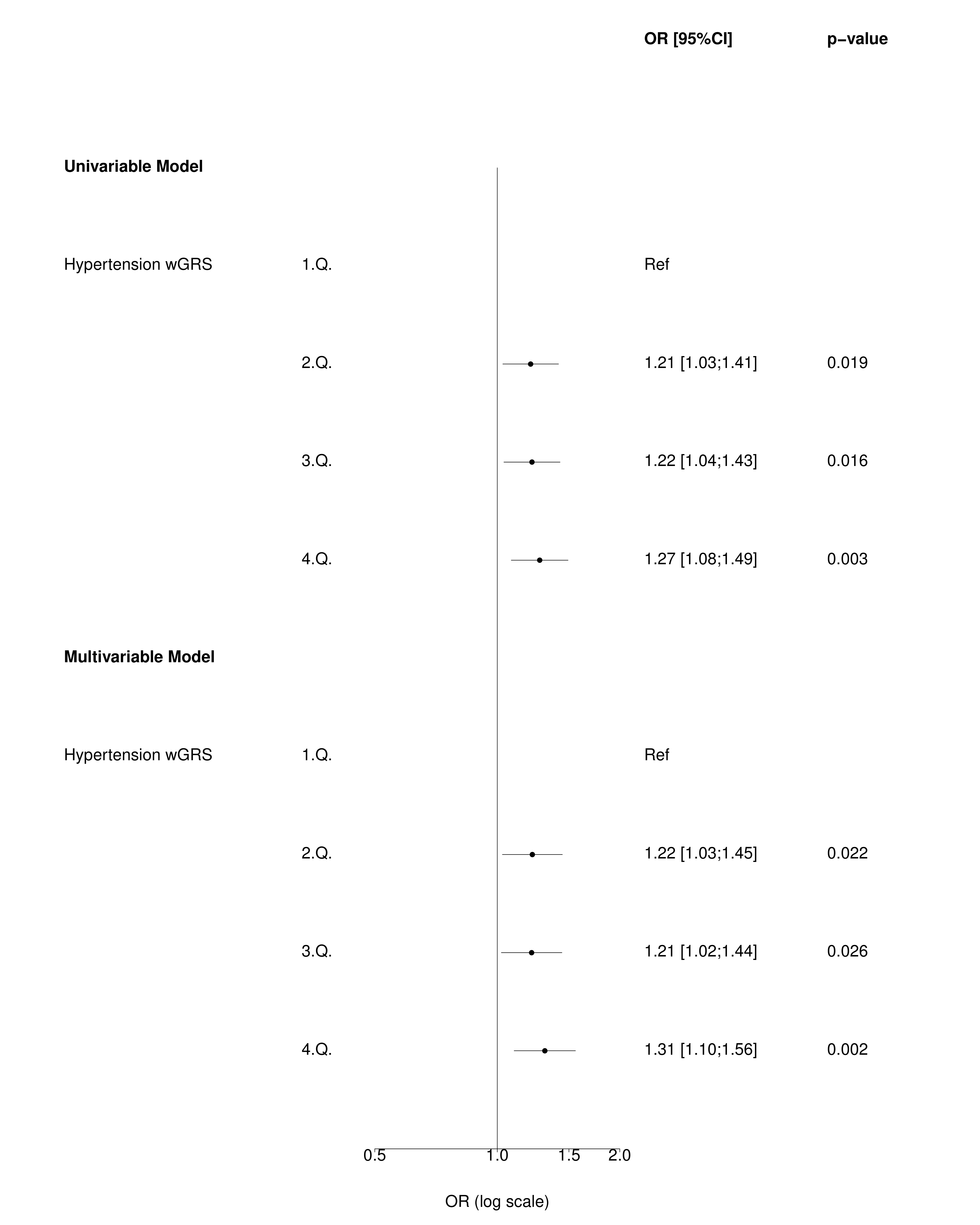

Supplement: S4 Fig — (TIFF) [file pone.0208645.s011.tiff]

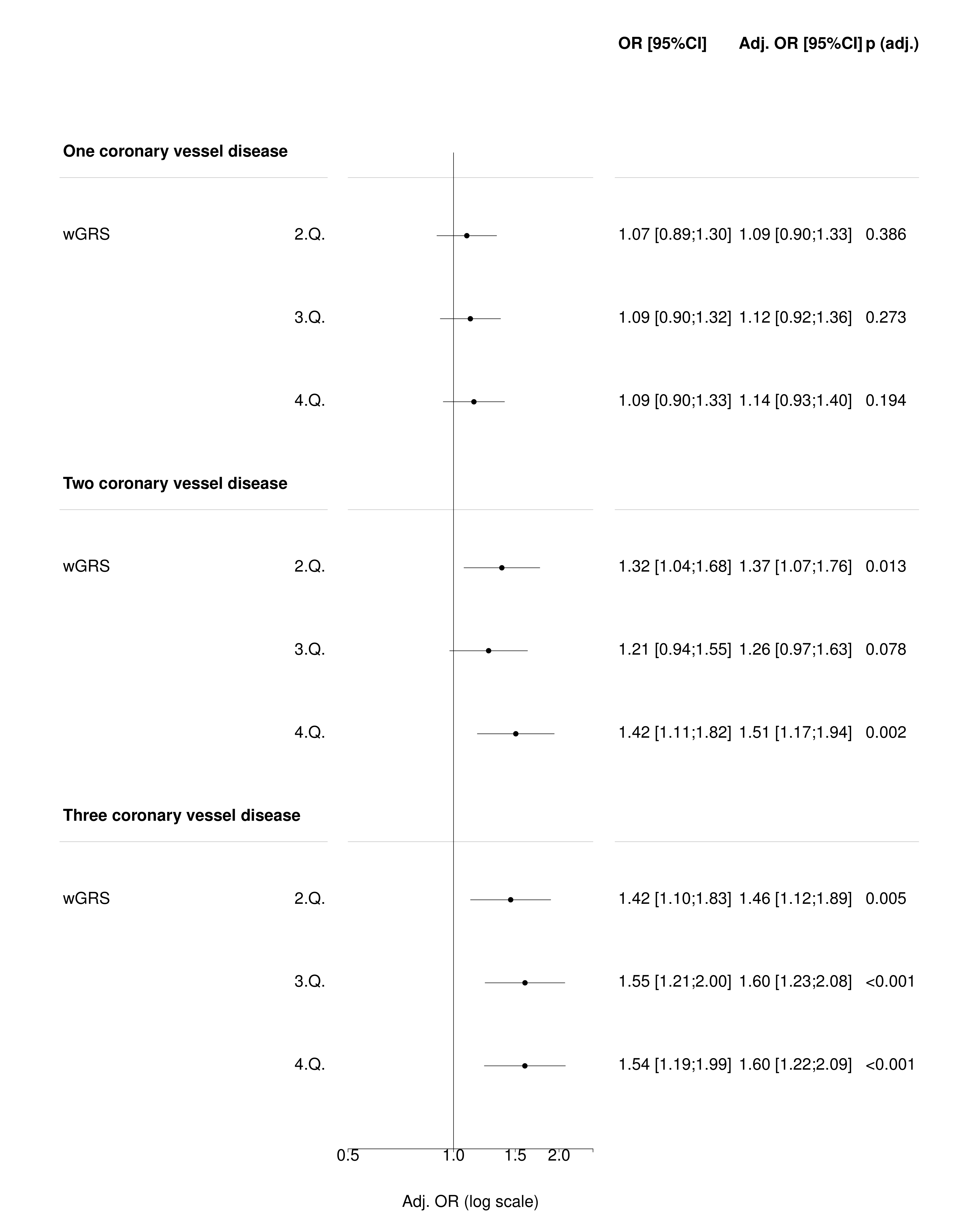

Supplement: S5 Fig — (TIFF) [file pone.0208645.s012.tiff]

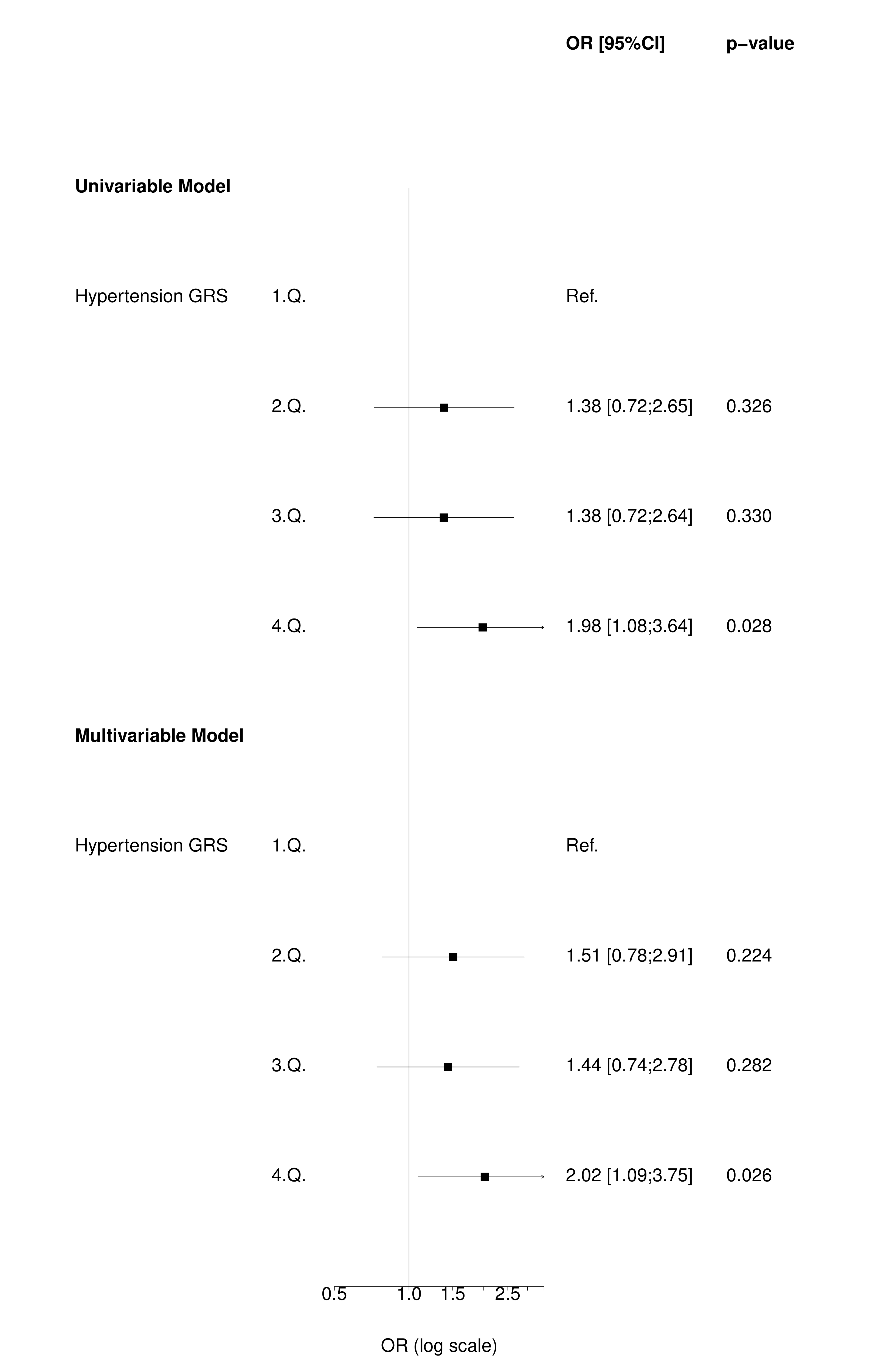

Supplement: S6 Fig — (TIFF) [file pone.0208645.s013.tiff]

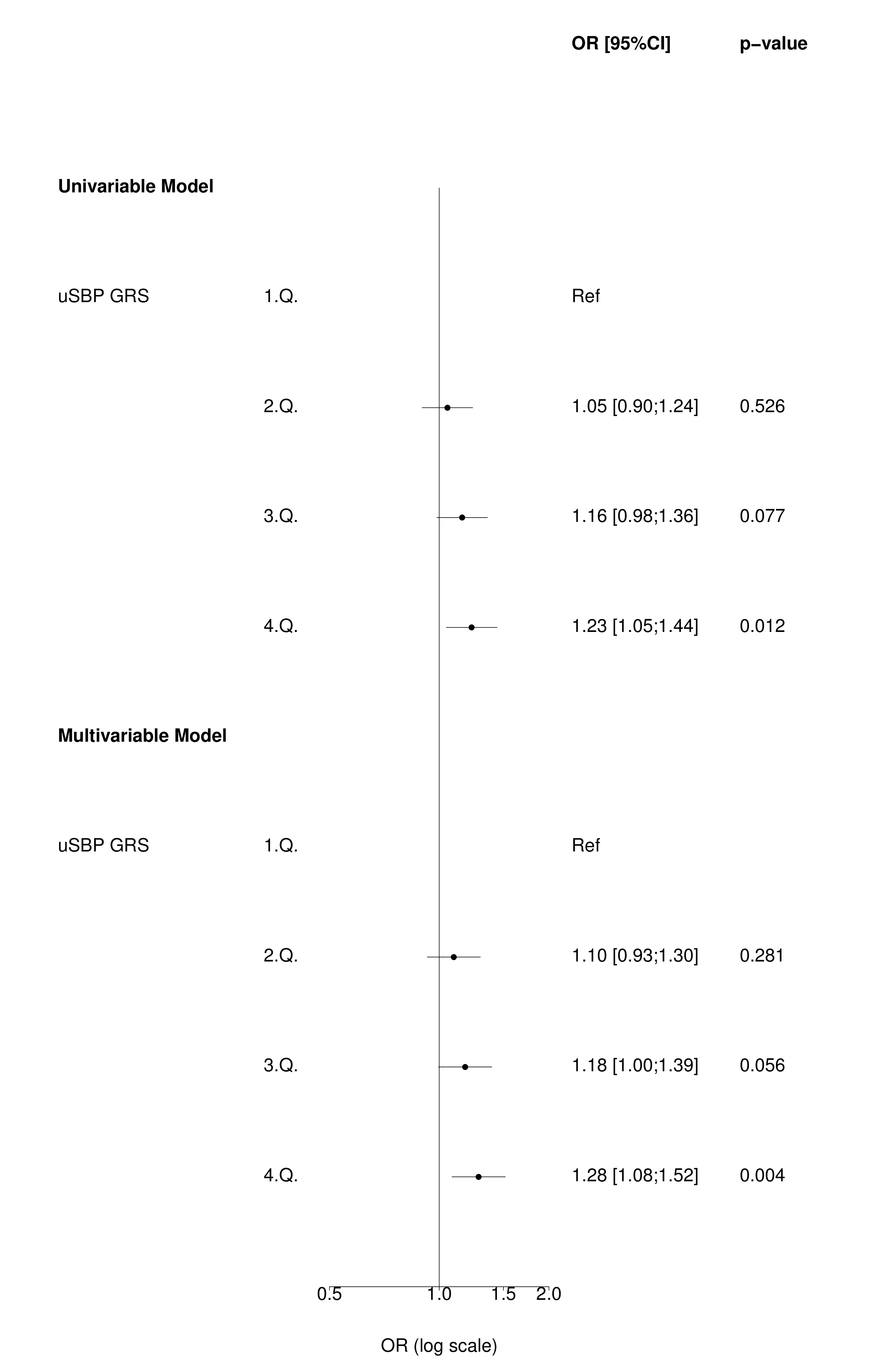

Supplement: S7 Fig — (TIFF) [file pone.0208645.s014.tiff]

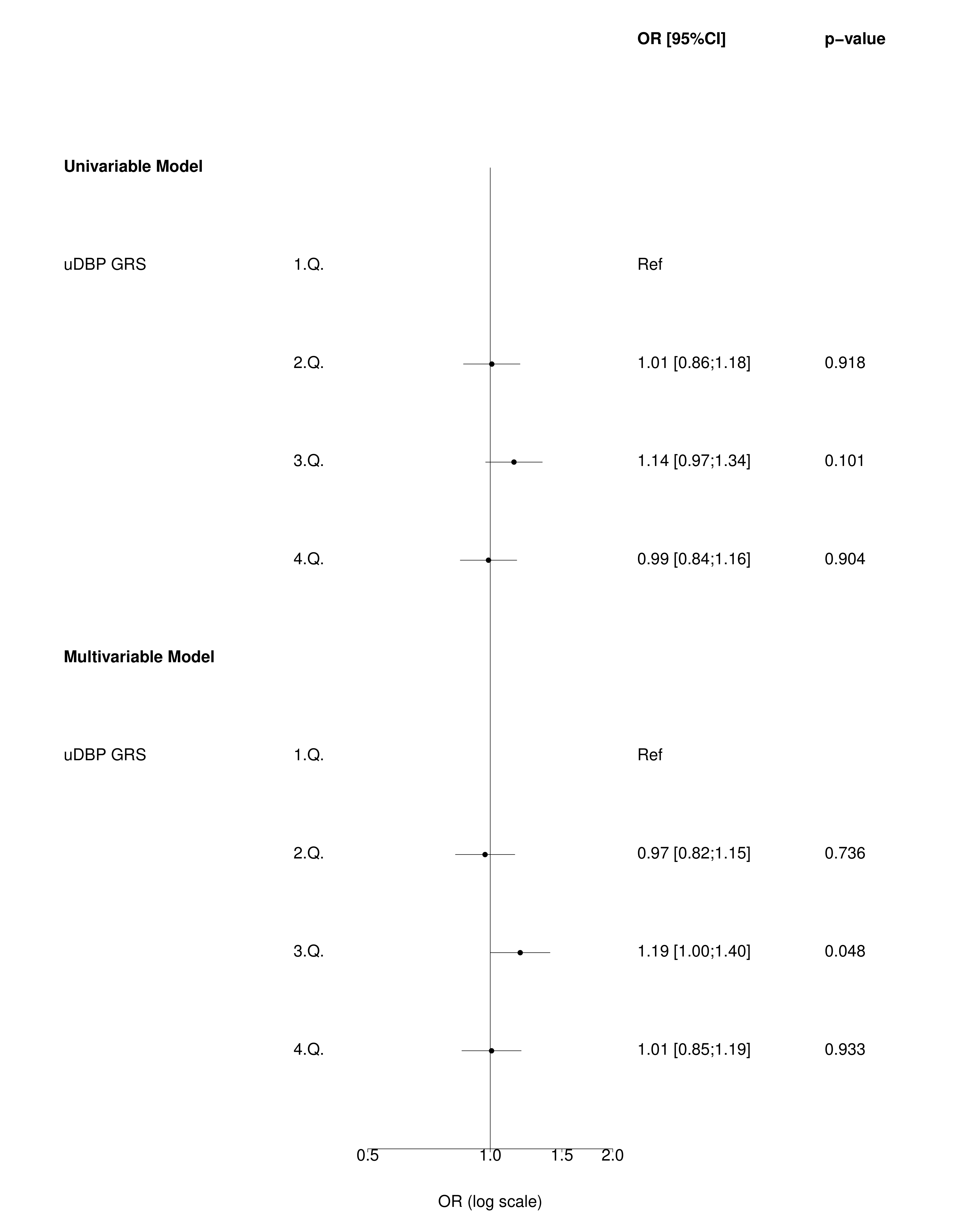

Supplement: S8 Fig — (TIFF) [file pone.0208645.s015.tiff]

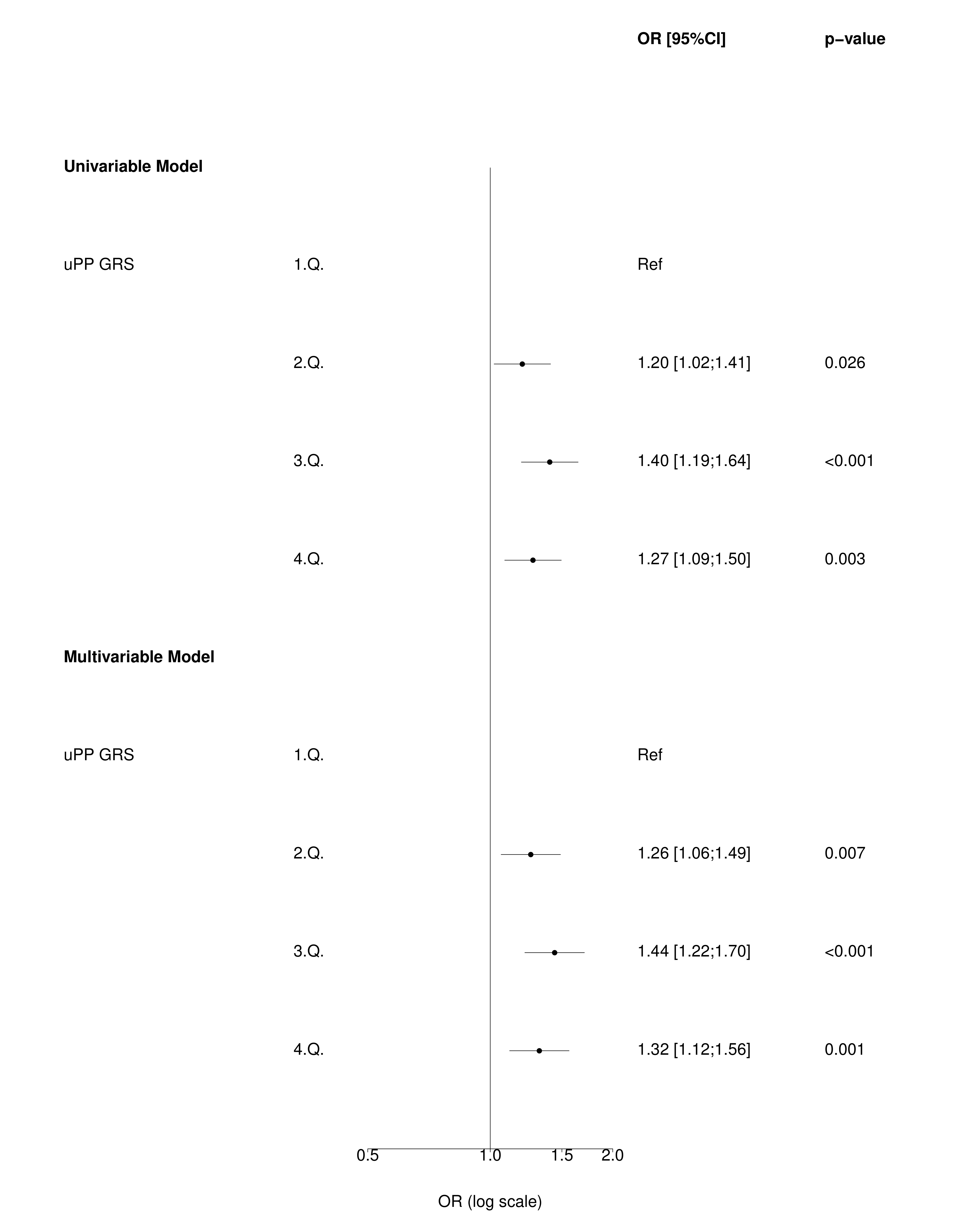

Supplement: S9 Fig — (TIFF) [file pone.0208645.s016.tiff]

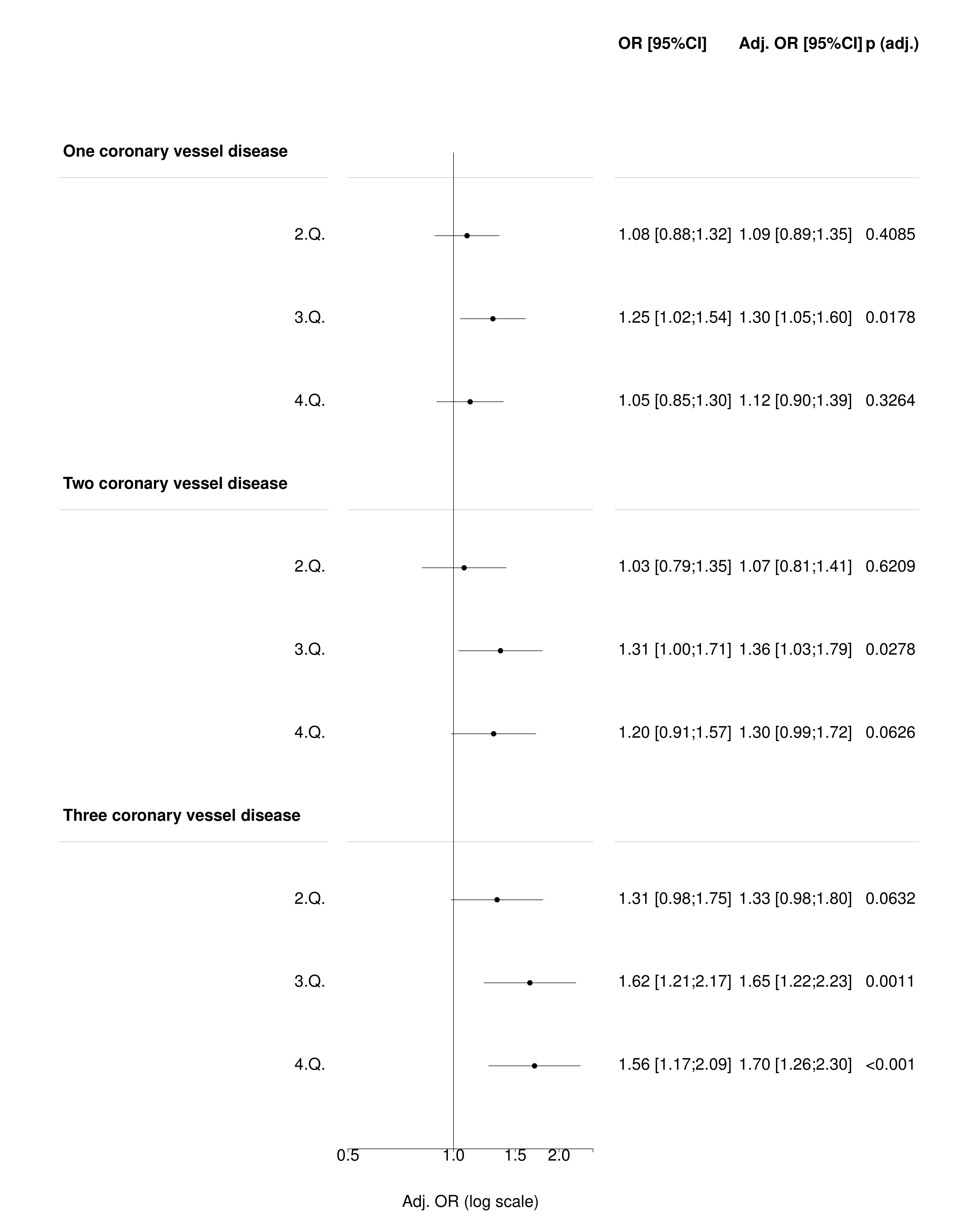

Supplement: S10 Fig — (TIFF) [file pone.0208645.s017.tiff]
